# Bright, photostable and long-circulating NIR-II nanoparticles for whole-process monitoring and evaluation of renal transplantation

**DOI:** 10.1093/nsr/nwad286

**Published:** 2023-11-08

**Authors:** Rongyuan Zhang, Ping Shen, Yu Xiong, Tianjing Wu, Gang Wang, Yucheng Wang, Liping Zhang, Han Yang, Wei He, Jian Du, Xuedong Wei, Siwei Zhang, Zijie Qiu, Weijie Zhang, Zheng Zhao, Ben Zhong Tang

**Affiliations:** Clinical Translational Research Center of Aggregation-Induced Emission, The Second Affiliated Hospital, School of Medicine, School of Science and Engineering, Shenzhen Institute of Aggregate Science and Technology, The Chinese University of Hong Kong, Shenzhen (CUHK-Shenzhen), Shenzhen 518172, China; Center for AIE Research, Shenzhen Key Laboratory of Polymer Science and Technology, Guangdong Research Center for Interfacial Engineering of Functional Materials, College of Materials Science and Engineering, Shenzhen University, Shenzhen 518061, China; School of Chemistry, Xiangtan University, Xiangtan 411105, China; Center for AIE Research, Shenzhen Key Laboratory of Polymer Science and Technology, Guangdong Research Center for Interfacial Engineering of Functional Materials, College of Materials Science and Engineering, Shenzhen University, Shenzhen 518061, China; School of Chemistry, Xiangtan University, Xiangtan 411105, China; School of Chemistry, Xiangtan University, Xiangtan 411105, China; Clinical Translational Research Center of Aggregation-Induced Emission, The Second Affiliated Hospital, School of Medicine, School of Science and Engineering, Shenzhen Institute of Aggregate Science and Technology, The Chinese University of Hong Kong, Shenzhen (CUHK-Shenzhen), Shenzhen 518172, China; Clinical Translational Research Center of Aggregation-Induced Emission, The Second Affiliated Hospital, School of Medicine, School of Science and Engineering, Shenzhen Institute of Aggregate Science and Technology, The Chinese University of Hong Kong, Shenzhen (CUHK-Shenzhen), Shenzhen 518172, China; Center for AIE Research, Shenzhen Key Laboratory of Polymer Science and Technology, Guangdong Research Center for Interfacial Engineering of Functional Materials, College of Materials Science and Engineering, Shenzhen University, Shenzhen 518061, China; Clinical Translational Research Center of Aggregation-Induced Emission, The Second Affiliated Hospital, School of Medicine, School of Science and Engineering, Shenzhen Institute of Aggregate Science and Technology, The Chinese University of Hong Kong, Shenzhen (CUHK-Shenzhen), Shenzhen 518172, China; HKUST-Shenzhen Research Institute, Shenzhen 518057, China; Department of Chemistry, Hong Kong Branch of Chinese National Engineering Research Center for Tissue Restoration and Reconstruction, The Hong Kong University of Science and Technology, Hong Kong, China; Department of Urology, The First Affiliated Hospital of Soochow University, Suzhou 215006, China; Department of Urology, The First Affiliated Hospital of Soochow University, Suzhou 215006, China; Department of Chemistry, Hong Kong Branch of Chinese National Engineering Research Center for Tissue Restoration and Reconstruction, The Hong Kong University of Science and Technology, Hong Kong, China; Clinical Translational Research Center of Aggregation-Induced Emission, The Second Affiliated Hospital, School of Medicine, School of Science and Engineering, Shenzhen Institute of Aggregate Science and Technology, The Chinese University of Hong Kong, Shenzhen (CUHK-Shenzhen), Shenzhen 518172, China; Department of Urology, The First Affiliated Hospital of Soochow University, Suzhou 215006, China; Clinical Translational Research Center of Aggregation-Induced Emission, The Second Affiliated Hospital, School of Medicine, School of Science and Engineering, Shenzhen Institute of Aggregate Science and Technology, The Chinese University of Hong Kong, Shenzhen (CUHK-Shenzhen), Shenzhen 518172, China; HKUST-Shenzhen Research Institute, Shenzhen 518057, China; Clinical Translational Research Center of Aggregation-Induced Emission, The Second Affiliated Hospital, School of Medicine, School of Science and Engineering, Shenzhen Institute of Aggregate Science and Technology, The Chinese University of Hong Kong, Shenzhen (CUHK-Shenzhen), Shenzhen 518172, China; Department of Chemistry, Hong Kong Branch of Chinese National Engineering Research Center for Tissue Restoration and Reconstruction, The Hong Kong University of Science and Technology, Hong Kong, China; AIE Institute, Guangzhou 510530, China

**Keywords:** aggregation-induced emission, NIR-II imaging, kidney transplantation, nano-contrast agent, angiography

## Abstract

Kidney transplantation is the gold standard for the treatment of end-stage renal diseases (ESRDs). However, the scarcity of donor kidneys has caused more and more ESRD patients to be stuck on the waiting list for transplant surgery. Improving the survival rate for renal grafts is an alternative solution to the shortage of donor kidneys. Therefore, real-time monitoring of the surgical process is crucial to the success of kidney transplantation, but efficient methods and techniques are lacking. Herein, a fluorescence technology based on bright, photostable and long-circulating aggregation-induced emission (AIE) active NIR-II nano-contrast agent DIPT-ICF nanoparticles for the whole-process monitoring and evaluation of renal transplantation has been reported. In the aggregated state, DIPT-ICF exhibits superior photophysical properties compared with the commercial dyes IR-26 and IR-1061. Besides, the long-circulating characteristic of the AIE nano-contrast agent helps to achieve renal angiography in kidney retrieval surgery, donor kidney quality evaluation, diagnosing vascular and ureteral complications, and assessment of renal graft reperfusion beyond renovascular reconstruction, which considerably outperforms the clinically approved indocyanine green (ICG).

## Teaser

An AIE nano contrast agent with the longest absorption wavelength and highest absorbance in the AIE fluorophore family is designed and fabricated for monitoring kidney transplantation.

## INTRODUCTION

Medicine and surgery are the primary treatments for modern diseases. However, for some end-stage organ diseases, e.g. end-stage renal diseases (ESRDs), organ transplantation has been acknowledged as the most effective and life-saving treatment [1,2]. According to the Chinese Scientific Registry of Kidney Transplantation data, ∼11 000 patients with ESRD received a kidney transplant in 2020, while >60 000 patients remain on a waiting list for renal transplantation [3]. The dilemma of kidney source scarcity is also suffered by ESRD patients in other countries around the world [4,5]. In consideration of the organ shortage issue worldwide, improving the success rate of organ transplantation has become highly significant [6].

The kidney transplant procedure involves donor kidney retrieval (donor nephrectomy), donor kidney preservation, and vascular and ureteric reconstruction [7]. Among them, the quality of a donor kidney directly influences the outcome of the transplantation. Furthermore, renal revascularization and ureterovesical anastomosis also influence the reperfusion and urine excretion, respectively, playing a significant role in the success of renal transplantation surgery [8,9]. Hence, real-time evaluation and monitoring of kidney reperfusion and urine excretion before, during and after kidney implantation can greatly enhance the success rate of renal transplant surgery. Traditionally, the patency of vascular anastomosis was determined through visual inspection, assessing the arterial pulse quality, venous filling, color and turgidity of the renal graft [10]. However, this method heavily relied on the surgeons’ experience and often led to incorrect identification. While imaging techniques such as Doppler ultrasound, X-ray angiography [11], computed tomography (CT) [12], magnetic resonance imaging (MRI) [13] and radionuclide imaging [14] can be employed to diagnose surgical complications and assess renal graft reperfusion, most of these methods have limitations in real-time monitoring and tracing. Additionally, they may suffer from drawbacks such as radioactive exposure [15], high costs and cumbersome procedures. In terms of these, fluorescence imaging technology has emerged as an ideal approach for *in situ* and real-time monitoring of various diagnostic or therapeutic processes, such as cancer imaging in oncological surgeries, angiography and intraoperative identification of ureters [16–19]. Thus, employing fluorescence imaging technology to assess donor kidney reperfusion and monitor the patency of vascular anastomosis and ureterovesical connection offers a viable approach to enhance the success rate of renal transplantation surgery.

Fluorescence imaging technology strongly relies on imaging contrast agents. In particular, organic luminogens with absorption and emission properties in the second near-infrared region (NIR-II, 1000–1700 nm) are highly desirable contrast agents due to their excellent biocompatibility, deep penetration depth and high imaging contrast [20–22]. Despite the significant applications demonstrated by using organic NIR-II luminogens as fluorescent contrast agents, commonly used commercial imaging contrast agents, e.g. IR-26, IR-1061 and indocyanine green (ICG), face the challenge of concentration quenching [23]. This effect limits their utility to low concentrations, as higher concentrations can lead to diminished and even quenched emission. As a result, low concentration administration, poor photostability and rapid hepatobiliary clearance [24,25] markedly shorten the optimal imaging window of ICG (<5 min) [26]. The imaging duration is insufficient for guiding kidney transplant surgery, which commonly lasts 3.5–5.0 h [27]. Although, the circulating time and photostability of the molecular contrast agents could be improved by preparing their nanoaggregates, which mostly sacrificed the brightness of traditional planar luminogens including ICG, limiting their application for long-duration surgery guiding and monitoring. In this regard, NIR-II luminogens with high brightness, long-circulating time and high photostability in aggregates are highly desirable.

Aggregation-induced emission (AIE) luminogens (AIEgens) are unique luminescent materials with enhanced photoluminescence quantum efficiency and photostability in aggregated states [28]. These properties make them ideal candidates for preparing NIR-II emissive nano-contrast agents with high brightness and superior photostability. Notably, the brightness of a luminogen mainly depends on the production of molecular absorptivity and photoluminescence quantum yield (PLQY) [29]. The current molecular design of AIEgens often involves the introduction of highly twisted molecular rotors to prevent strong intermolecular interaction-caused luminescence quenching [30]. Unfortunately, this strategy typically leads to a relatively lower molar absorptivity. Therefore, designing AIE luminogens with both high molar absorptivity and high PLQY remains a significant challenge.

**Scheme 1.**
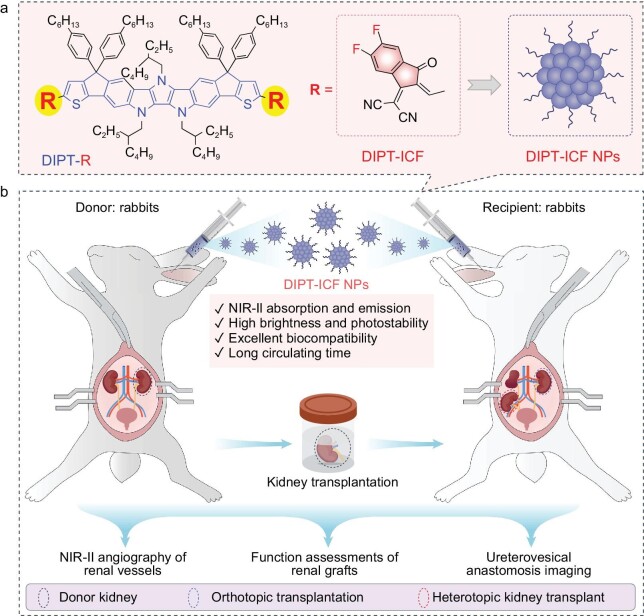
The development of the NIR-II nano-contrast agent (DIPT-ICF NPs) for monitoring the whole process of kidney transplantation. (a) Schematic illustration of the chemical structures of DIPT-IC and DIPT-ICF, as well as the aggregation state of DIPT-ICF. (b) Scheme of the application of DIPT-ICF NPs for monitoring and evaluation of multiple processes in kidney transplantation.

Fused-ring electron acceptors (FREA) for organic solar cell applications have been rapidly developed in recent years. These materials have absorption wavelengths mainly located in the NIR region, providing superior light-harvesting capability [31]. However, the luminescence properties of these materials have scarcely been explored and applied in the biomedical field. In this work, by learning from the molecular design of FREA, we designed and synthesized two-expanded molecules, DIPT-IC and DIPT-ICF, with rigid and planar conjugation structures. The results demonstrate that, compared with DIPT-IC, DIPT-ICF exhibits longer absorption and emission wavelengths, along with superior AIE properties, featuring higher molar absorptivity (molar extinction coefficient (MEC)) and higher PLQYs. These properties grant them high brightness in aggregates when compared with commercial dyes such as IR-26 and IR-1061. In view of this, the water-dispersible nanoparticle of DIPT-ICF thus was prepared (Scheme 1a) and applied in the monitoring and evaluation of the entire process of kidney transplantation (Scheme 1b). Our findings indicate that a single dose of DIPT-ICF nano-contrast agent nanoparticles (NPs) could clearly monitor the entire surgical process via luminescence, outperforming the clinically approved contrast agent ICG. The study underscores the potential of fluorescent imaging technology using NIR-II contrast agents to monitor the entire organ transplantation surgery process and diagnose potential surgical complications. This technology could serve as a reliable tool for transplant surgeons, thereby enhancing the success rate of organ transplantation.

## RESULTS AND DISCUSSION

### Synthesis and characterization

First, two-expanded acceptor–donor–acceptor molecules, DIPT-IC and DIPT-ICF, have been designed and synthesized. The synthetic routes are shown in Supplementary Scheme S1. Compound **1** was synthesized according to the literature method [32]. Suzuki coupling between compound **1** and methyl 2-bromothiophene-3-carboxylate gives intermediate **2**. Intermediate **2** further reacts with 1-bromo-4-hexylbenzene in the presence of n-BuLi, affording fused-ring intermediate **3**. Compound **3** undergoes a Vilsmeier–Haack reaction and produces DIPT-CHO. Finally, a Knoevenagel reaction between DIPT-CHO and Indanone-cyano derivatives affords the two targeting molecules DIPT-IC and DIPT-ICF. All the intermediates and target molecules have been characterized by using ^1^H NMR, ^13^C NMR and Mass spectra (Supplementary Figs S1–S15).

**Figure 1.**
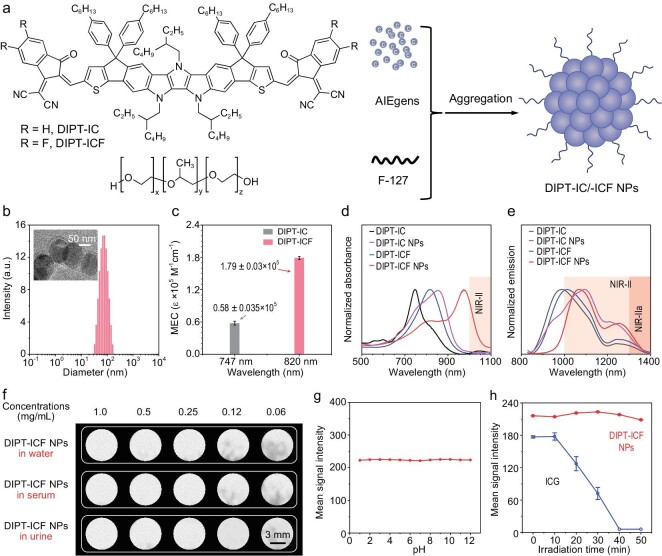
Preparation and characterization of DIPT-IC, DIPT-ICF, DIPT-IC NPs, and DIPT-ICF NPs. (a) Chemical structures of DIPT-IC, DIPT-ICF and amphiphilic polymer F127, and schematic illustration for the designs of DIPT-IC NPs and DIPT-ICF NPs. (b) Dynamic light scattering data and transmission electron microscopy image (the scale bar of the inset indicates 50 nm) of DIPT-ICF NPs. (c) Molar absorptivity of DIPT-IC and DIPT-ICF. (d) Absorption spectra of DIPT-IC and DIPT-ICF solutions (in THF) and their NPs in deionized water. (e) Emission spectrum of DIPT-IC and DIPT-ICF in THF and their NPs in deionized water. (f) NIR-II images of DIPT-ICF NPs in deionized water, serum and urine with different concentrations (1, 0.5, 0.25, 0.125 and 0.0625 mg mL^−1^) using a 980-nm laser as the excitation source with a power density of 60 mW cm^−2^ (the scale bar indicates 3 mm). (g) Mean signal intensity of DIPT-ICF NPs in the NIR-II window at different pH states. (h) Photostability measurements of DIPT-ICF NPs and ICG upon irradiation with an 808-nm laser with a time interval of 10 min at a power density of 100 mW cm^−2^.

Following that, the photophysical properties of DIPT-IC, DIPT-ICF and their NPs were investigated. The DIPT-IC NPs and DIPT-ICF NPs were prepared using the nanoprecipitation method with the FDA-approved surfactant F-127 (Fig. 1a). We utilized dynamic light scattering (DLS) and transmission electron microscopy (TEM) to analyse their hydrated particle sizes and morphology, respectively (Fig. 1b and Supplementary Fig. S16). Both DIPT-IC and DIPT-ICF showed strong absorption in tetrahydrofuran (THF) solution with maximal absorption peaks of 737 and 820 nm, respectively. Compared with the non-fluorinated molecule DIPT-IC, the absorption of the fluorinated molecule DIPT-ICF showed a red shift of 83 nm, which could be ascribed to the enhanced intramolecular charge-transfer effect induced by the electron-withdrawing fluorine atoms [33]. Density functional theory (DFT) calculation revealed that DIPT-ICF displayed a smaller energy gap than DIPT-IC (1.844 vs 1.879 eV), which matched well with the absorption data. Moreover, the greatly overlapped highest occupied molecular orbital and lowest unoccupied molecular orbital electron density distribution suggests favorable electron transition, which gives the benefit of a high molar absorptivity (Supplementary Fig. S17). Indeed, the MEC at 820 nm for DIPT-ICF is remarkably high at 1.79 × 10^5^ M^−1^ cm^−1^, which is 3-fold higher than that of DIPT-IC (0.58 × 10^5^ M^−1^ cm^−1^, 737 nm), suggesting that the introduction of a fluorine atom can also improve the molar absorptivity (Fig. 1c and Supplementary Figs S18 and S19). Hence, the incorporation of fluorine atoms contributes to both the red shift in absorption and the enhancement of molar absorptivity. It is worth mentioning that such a high molar absorptivity will contribute to the preparation of high-brightness nano-contrast agents [34]. Additionally, in the aggregated state, the absorption spectra of DIPT-ICF NPs generates a further bathochromic shift compared with its single-molecule state, which suggests the formation of aggregate such as J-aggregation [35]. Especially, the absorption peak of DIPT-ICF NPs can even shift to 974 nm with the tail extending to 1100 nm, which is favorable for achieving a deep penetration depth for *in vivo* imaging (Fig. 1d).

Next, we investigated the photoluminescence (PL) properties of DIPT-IC and DIPT-ICF in both the aggregated and single-molecule states. As shown in Fig. 1e, the emission peaks of DIPT-IC and DIPT-ICF were measured at 988 and 1014 nm, respectively. Upon aggregation, the emission of their NPs red-shifted to 1090 and 1078 nm, respectively. The strongly interacted molecules within the aggregates will result in the energy split and generate a lower first excited state relative to the monomer. According to Kasha's rule, the excitons mostly will go back to the lowest excited state energy level through internal conversion and then go back to the ground state through radiative decay. This can explain why the aggregates usually exhibit more red-shifted emissions than the monomer. Notably, both DIPT-IC and DIPT-ICF exhibited shoulder peaks that extended to the NIR-IIa region (1300–1400 nm), which were much favorable to *in vivo* imaging. The absolute PLQY of DIPT-ICF (0.09%) was 1.5 times higher than that of DIPT-IC (0.06%), surpassing the commercial NIR-II dyes IR-26 (0.05% [36]) and IR-1061 (0.08%) (Supplementary Table S1). As a result, the brightness of DIPT-ICF (161.1 M^−1^ cm^−1^) is 4.7 and 2.2 times higher than that of DIPT-IC (34.62 M^−1^ cm^−1^) and IR-26 (74.5 M^−1^ cm^−1^), respectively, owing to the brightness being from the multiplication of MEC and PLQY [37]. Although the IR-26 molecule possesses longer absorption and emission wavelengths compared with DIPT-ICF, its inherent aggregation-caused quenching effect renders it non-luminescent in the aggregated state. Similarly to IR-26 NPs, no NIR-II fluorescence was detected for IR-1061 NPs (Supplementary Figs S20 and S21, and Supplementary Table S1). Thanks to the AIE properties of DIPT-ICF, DIPT-ICF NPs still exhibit remarkably high brightness (Supplementary Fig. S22 and Supplementary Table S1). These findings indicate that DIPT-ICF is more suited for developing nano-contrast agents for application in physiological environments.

### Fluorescence stability, depth penetration, biosafety and angiographic parameters assessment

The outstanding optical properties of DIPT-ICF NPs further inspire us to explore their imaging performance as a fluorescent contrast agent. Figure 1f and Supplementary Fig. S23a show that the fluorescence signal of DIPT-ICF NPs was gradually enhanced when the concentrations and afforded stable fluorescent signals were increased in different aqueous media, suggesting the superior stability of the fluorescent nano-contrast agent in the body fluid environment. Since the pH value of interstitial fluid varies with organ metabolism, the PL intensity of DIPT-ICF NPs under different pH was also investigated. It was almost unchanged when varying the pH from 1 to 12 in phosphate buffered solution (PBS), demonstrating the high stability of DIPT-ICF NPs against pH variations (Fig. 1g and Supplementary Fig. S23b). Photostability of the imaging contrast agent is also significant for biological process monitoring or guiding the surgery. A contrast agent with good photostability can prevent the photobleaching effect during imaging, ensuring the accuracy of visualization. Under continuous laser irradiation (808 or 980 nm), DIPT-ICF NPs demonstrated superior photostability in comparison with ICG in an aqueous environment (Fig. 1h and Supplementary Figs S23c and S24). This property is advantageous for continuous imaging events and long-circulating fluorescence angiography applications.

Before applying DIPT-ICF NPs for *in vivo* experimentation, the penetration depth and imaging resolution of DIPT-ICF NPs were evaluated *in vitro* through using tissue phantoms [38]. As shown in Supplementary Fig. S25a, pre-covering DIPT-ICF NPs filled capillary tubes with chicken breast tissues and the signal background ratios (SBRs) of NIR-I and NIR-II imaging were measured. Fluorescence signals from the capillary tubes were clearly detected in both the NIR-I and the NIR-II channels when the thickness of the chicken breast tissue was <2 mm (Supplementary Fig. S25b and c). At a tissue thickness of 3 mm, NIR-II imaging demonstrates its superior penetration depth, exhibiting significantly higher SBRs compared with NIR-I imaging (Supplementary Fig. S25d).

Next, prior to assessing the suitability of DIPT-ICF NPs for *in vivo* angiography, their biocompatibility and biosafety were thoroughly evaluated. DIPT-ICF NPs do not exhibit a significant dark toxicity or photodynamic or photothermal effect at low energy density (∼100 mW cm^−2^), ensuring that there is no harm to cells and tissues during the imaging process (Supplementary Figs S26–S31 and Supplementary Tables S2 and S3). Accordingly, DIPT-ICF NPs is a highly biocompatible and biologically safe contrast agent that can be used in mice and rabbits. In the subsequent experiment, subcutaneous fluorescence angiography was conducted on the abdomens of the mice using various laser filter combinations, including a 808-nm laser paired with a 900-nm long pass filter (LP) and a 1319-nm LP, as well as a 980-nm laser combined with a 1020-nm, 1100-nm and 1319-nm LP, respectively (Supplementary Fig. S32a and b). These results showed that employing a 980-nm laser for excitation and a 1319-nm LP filter yielded angiography images with superior resolution and higher SBRs. This enhancement can be attributed to the increased brightness and deeper penetration depth in this specific wavelength range (Supplementary Fig. S32c and d). Consequently, for the subsequent angiography experiments, a 980-nm laser and a 1319-nm LP filter were utilized.

### Assessment of blood retention time and optimal time-window duration of angiography

**Figure 2.**
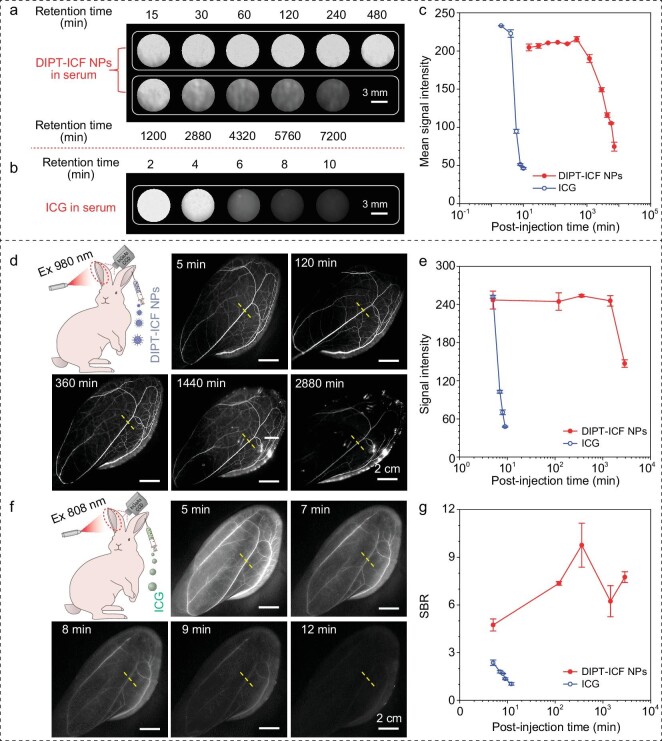
Comparison of blood retention time and optimal time-window duration for angiography using DIPT-ICF NPs and ICG as contrast agents, respectively. (a) NIR-II images of serum samples collected from rabbits at different time points (15, 30, 60, 120, 240, 480, 1200, 2880, 4320, 5760 and 7200 min) post-injection of DIPT-ICF NPs through ear veins were captured (980-nm laser, 60 mW cm^−2^, 1319-nm LP and exposure time 200 ms). (b) NIR-II images of serum samples collected from rabbits at different time points (0, 2, 4, 6, 8 and 10 min) after injection of ICG. (c) The quantitative analysis curves of the mean fluorescence intensity in (a) and (b). (d) NIR-II angiograms of rabbit ears were performed at 5, 120, 360, 2880 and 4320 min after injection of DIPT-ICF NPs. (e) Curves of fluorescence intensity versus time of blood vessels marked with yellow dashed lines in (d) and (f). (f) The NIR-I angiography was performed in rabbit ears at 5, 7, 8, 9 and 12 min after injection of ICG. (g) Quantitative analysis indicated that NIR-II angiography in (d) using DIPT-ICF NPs as a fluorescence contrast agent had a significantly higher SBR than that of NIR-I angiography in (e) using ICG.

To achieve the whole-process monitoring and evaluation of renal transplantation through the fluorescence technique, a long circulating time of contrast agents is necessary. Initially, the DIPT-ICF NPs and ICG were injected intravenously into mice (0.3 mg mL^−1^ in PBS, 200 μL per mouse) and rabbits (0.3 mg mL^−1^ in PBS, 0.8 mg kg^−1^). Serum samples were then collected at various time points to record the luminescence signals. Remarkably, strong NIR-II signals of DIPT-ICF NPs could be detected from the serum samples of mice and rabbits even after 1440 (24 h) and 2880 (48 h) min of circulation (Supplementary Fig. S33a and Fig. 2a). In stark contrast, the ICG signal in serum samples decayed rapidly, indicating a short circulating retention time (Supplementary Fig. S33b and Fig. 2b). Quantitative analysis of the signal intensity in serum samples from both mice and rabbits revealed that the half-life of DIPT-ICF NPs in blood circulation significantly exceeded that of ICG (Supplementary Fig. S33c and Fig. 2c). These results validated the long retention time of DIPT-ICF NPs in the blood circulation of live animals.

Subsequently, an evaluation of the optimal time window for angiography was conducted. Angiography of subcutaneous vasculature in the abdomen of mice and auricles of rabbits showed that stable and strong luminescence of DIPT-ICF NPs was clearly observed even beyond 720 min (12 h) (Supplementary Fig. S33d) and 2880 (48 h) (Fig. 2d and e) after intravenous injection, respectively. In contrast, the signals from ICG rapidly attenuated on the abdomens of mice and ears of rabbits after injection, within 20 and 9 min, respectively (Supplementary Fig. S33e and Fig. 2f). Additionally, the SBRs, defined as the vessel-signal-to-skin ratios, in the angiograms utilizing DIPT-ICF NPs as a contrast agent were significantly higher than those of ICG (Supplementary Fig. S33f and Fig. 2g). These results clearly demonstrate that DIPT-ICF NPs as a contrast agent were more suitable for fluorescence angiography compared with the commercial dye ICG. The exceptional angiographic performance of DIPT-ICF NPs motivates us to further explore its real-world applications for in-surgical renal vessels angiography and evaluation of renal graft reperfusion in rabbit models.

### Renovascular angiography and perfusion assessment in the donor and recipient rabbit models

**Figure 3.**
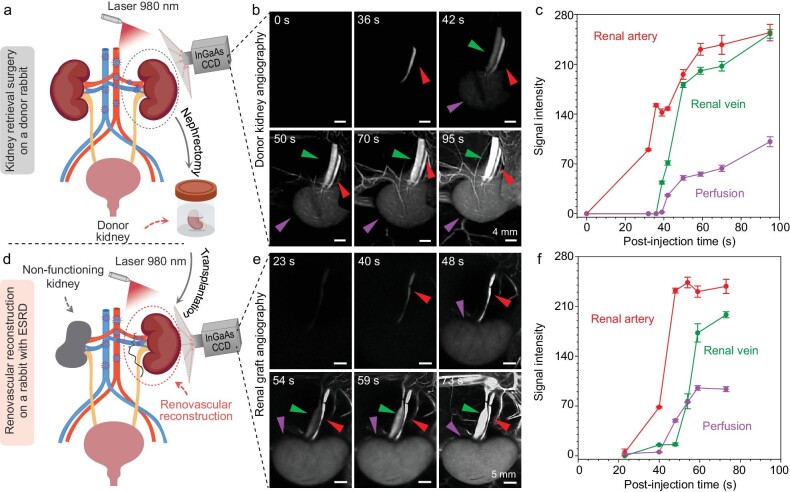
Renal angiography of donor and recipient rabbits in the NIR-II window based on DIPT-ICF NPs during kidney retrieval and implantation, respectively. (a) A schematic of the donor kidney retrieval surgery and renal angiography on a donor rabbit. (b) NIR-II images of renal arteries, renal veins and donor kidneys at different time points after injection of DIPT-ICF NPs via rabbit ear veins. (c) Quantitative analysis of NIR-II fluorescence intensity of kidneys and their accessory vessels conducted in (b). (d) A schematic of the kidney implantation surgery and renal angiography after revascularization. (e) NIR-II images of graft arteries, veins and graft reperfusion were captured at various time points following renal vessel anastomosis after intravenous administration of DIPT-ICF NPs. During kidney transplantation, the renal graft artery, vein and the renal graft reperfusion could be visualized in real time with high resolution and the patency of the anastomosis of the graft vascular could be observed by using NIR-II imaging. (f) Quantitative analysis of NIR-II fluorescence intensity of the renal graft artery, the renal graft vein and the renal graft reperfusion that was conducted in (e). Red arrows, green arrows and purple arrows represent renal arteries, renal veins and kidneys. All NIR-II images were taken using a 980-nm laser at a power density of 60 mW cm^−2^, a 1319-nm LP filter and an exposure time of 200 ms.

Precise anatomy of the renal vasculature and accurate assessment of renal perfusion are greatly significant for kidney transplantation. To evaluate the efficacy of DIPT-ICF NPs in renovascular angiography, we successfully established donor kidney nephrectomy and orthotopic kidney transplantation models to simulate clinical scenarios. As illustrated schematically in Fig. 3a, after the successful establishment of rabbit models, we performed NIR-II renal angiography following the intravenous administration of the AIE nano-contrast agent. Upon injection, the renal artery and vein were illuminated at 32 and 39 s, respectively. The kidney outline became clearly visible at 42 s and significantly intensified at 95 s (Fig. 3b). The high-resolution renovascular angiography revealed a single renal artery and vein in the donor rabbit without malformations (Fig. 3b, red and green arrows). The fluorescence signal was uniformly distributed over the donor kidney surface, suggesting good renal perfusion (Fig. 3b, purple arrows). Quantitative analysis of intensity over time confirmed unobstructed blood flow in both the renal artery and vein, indicating that the donor kidney met the transplantation criteria (Fig. 3c).

Subsequently, the donor kidney was orthotopically transplanted into another rabbit (Fig. 3d). After successful revascularization of the renal graft, renovascular angiography was performed. As depicted in Fig. 3e, the reconstructed graft artery and vein were sequentially visualized from 40 to 54 s post-intravenous injection of DIPT-ICF NPs. The anastomosis sites on the renal artery and vein were clearly observed, with no other NIR-II signals detected in the surgical area, indicating proper blood flow within the reconstructed vessels. In addition, the luminescence in the NIR-II region is evenly distributed on the grafted kidney, which is attributed to the high patency of the anastomosis sites that can afford good blood perfusion to the transplanted kidney. Quantitative analysis of the NIR-II signal from reconstructed vessels showed a similar tendency to change to that of renovascular angiograms on the donor rabbit (Fig. 3f). These results indicate the successful achievement of vascular anastomosis during renal transplantation.

It is worth noting that, due to the high brightness and deep penetration depth of DIPT-ICF NPs, even the renal vein concealed beneath soft tissues could be clearly and easily distinguished. This capability enables precise ligation, which is crucial for averting significant hemorrhage in live donors. Moreover, precise localization and swift separation of renal vessels during donor nephrectomy enhance the efficiency of kidney preparation prior to the transplant procedure. As shown in Supplementary Fig. S34, the two branches of the left renal vein (one venule of ∼1.0 mm in diameter and one cranial abdominal vein of ∼2.5 mm in diameter [39]) could be easily and effectively ligated and severed using the image-guided donor nephrectomy. These results demonstrated the feasibility of DIPT-ICF NPs to assist NIR-II renal angiography for guiding kidney transplantation. Such technology could be invaluable for surgeons in promptly discovering and addressing surgical complications.

### NIR-II angiography for diagnosis of vascular complications and assessment of graft reperfusion in orthotopic kidney transplantation in rabbit models

**Figure 4.**
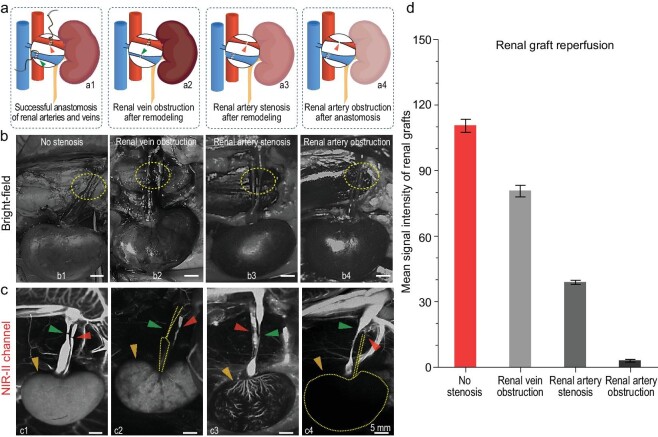
Application of NIR-II angiography for real-time monitoring of renovascular anastomosis during orthotopic kidney transplantation on rabbit models. (a) Schematic illustration of possible vascular complications, e.g. anastomotic stenosis and obstruction of renal vessels after renovascular reconstruction. (b) The bright-field and (c) NIR-II angiograms of different vascular complications. (d) Mean NIR-II intensity measurements of renal grafts in (c). The red, green and orange arrows in (c) represent the arteries, veins and blood reperfusion of renal grafts. All NIR-II images were captured using a 980-nm laser at a power density of 60 mW cm^−2^, a 1319-nm LP filter and an exposure time of 200 ms.

Anastomotic stenosis is a common complication that could directly cause hypoperfusion of the allograft and lead to delayed graft function (DGF) [40]. However, these complications are often challenging to identify using the naked eye. To assess the feasibility of DIPT-ICF NPs in rapidly diagnosing anastomotic failure in renal graft vasculature, three models of anastomotic stenosis in orthotopic kidney transplantation were developed (Fig. 4a1–4). Following the intravenous administration of DIPT-ICF NPs into rabbit models, the patency of the renal vessels and reperfusion of the renal graft were imaged after revascularization. As shown in Fig. 4b1–4, hardly any abnormalities at the vascular anastomosis sites or on the graft perfusion status were identified using the naked eye. However, in the NIR-II channel, successful revascularization without any stenosis displayed bright fluorescence along vascular routes (Fig. 4c1, green and red arrows). Meanwhile, the NIR-II fluorescence distributed uniformly on the kidney grafts (Fig. 4c1, orange arrow). Despite the occlusion at the anastomotic site of the renal vein (Fig. 4c2, green arrow), a significant amount of blood continued to flow into the renal graft due to the unobstructed blood flow in the renal artery (Fig. 4c2, red arrow). Consequently, the NIR-II fluorescence signal can still be detected in the transplanted kidney (Fig. 4c2, orange arrow). In cases in which the renal artery was partially narrowed at the anastomosis site, the arterial resistance increased, causing reduced blood flow into the distal renal artery (Fig. 4c3, red arrow). As a result, poor renal graft reperfusion emerged (Fig. 4c3, orange arrow). When the anastomotic site of the renal artery was completely blocked (Fig. 4c4, red arrow), no blood perfused into the transplanted kidney and hence no fluorescence signal could be detected from the kidney (Fig. 4c4, orange arrow). These abnormalities could be distinctly presented in real time to surgeons via the AIE contrast agent-based NIR-II angiograms. Further quantitative analyses of the fluorescence obtained from the reconstructed renal vessels and renal grafts confirmed that the abnormal vascular anastomosis was accompanied by poor renal reperfusion (Fig. 4d).

To create a more realistic simulation of vascular anastomosis in kidney transplantation, heterotopic kidney transplant models in rabbits were established. Prior to the donor kidney implantation, the iliac vessels were identified and assessed regarding suitability for anastomosis. Based on the colorful image shown in Supplementary Fig. S35a, distinguishing the iliac vessels and their branches from the complex vascular networks against the blood-red background was challenging. Without precise separation of the iliac vasculatures during the operation, iatrogenic obturator nerve injury and serious complications could occur. Fortunately, the AIE nano-contrast agent, with strong brightness and long-circulating properties, provided high-resolution and prolonged angiograms in the NIR-II channel, illuminating the iliac vessels, including the iliac artery and vein, as well as the vascular branches of the postcava and ventral aorta.

Once the anatomy of the iliac vessels was clearly demonstrated, ectopic renal implantation was performed involving an end-to-side anastomosis of the graft artery and the common iliac artery, and an end-to-end anastomosis of the renal vein and common iliac vein [7]. As shown in the NIR-II channel of Supplementary Fig. S35b, the patency of the vascular anastomosis and reperfusion of the renal allograft were clearly displayed. Overall, abnormal anastomosis of graft vasculatures and the resulting poor renal allograft reperfusion could be easily diagnosed through DIPT-ICF NPs-based NIR-II angiography.

### NIR-II imaging of ureterovesical junction in kidney transplantation

Ureterovesical anastomosis in kidney transplantation is another main step in the surgery [41]. However, surgical errors and ureteral ischemia resulting in necrosis can lead to ureteral complications such as urinary leakage (with an incidence of ∼9.3%) and ureteral stenosis (with an incidence of ∼10.5%) at the site of the ureteral junction (Supplementary Fig. S36a), posing a significant threat to graft function [42,43]. The remarkable *in vivo* imaging capabilities of DIPT-ICF NPs make them ideal for diagnosing ureteral complications caused by anastomotic failure. By injecting DIPT-ICF NPs (0.1 mg mL^−1^, dispersed in PBS solution) into the ureter anterogradely via the renal pelvis, both the ureter and the bladder were immediately illuminated.

As shown in Supplementary Fig. S36b and c, in a successful anastomosis model, no urinary leakage or obstruction was observed at the anastomotic site and the bladder could be successfully visualized. Conversely, in the obstruction model of ureterovesical anastomosis, the obstruction prevented the recipient's bladder being filled with the DIPT-ICF NPs, leading to the absence of a fluorescent signal below the anastomotic site of the ureter and bladder (Supplementary Fig. S36d). Furthermore, the bright fluorescence signal around the recipient's bladder indicated urine leakage from the ureterovesical suturing site (Supplementary Fig. S36e). Quantitative analysis revealed that the mean fluorescence intensity of the recipient's bladder in both the urinary leak and the ureteral stenosis models was significantly reduced compared with the successful urinary tract reconstruction model, indicating that urine excreted by the donor kidney could not flow unimpeded into the bladder (Supplementary Fig. S36f). These findings highlight the capability of DIPT-ICF NPs as an outstanding contrast agent for fluorescent ureterography in the NIR-II window.

### Donor kidney quality assessment prior to implantation via NIR-II imaging based on DIPT-ICF nano-contrast agent

Good donor kidney quality is essential for long-term graft survival [44]. A rapid and non-invasive assessment of donor kidney quality is crucial before implantation. To our knowledge, the use of NIR-II imaging technology to assess the quality of donor kidneys before renal implantation has been rarely reported. Thrombosis in the donor kidney directly impacts graft survival after kidney transplantation. Therefore, we initially established two intrarenal thrombosis models by flushing preservation fluids containing non-degradable hydrogel microspheres (200 and 500 μm in diameter) into the donor kidneys. As shown in Supplementary Fig. S37, after perfusing DIPT-ICF NPs through the renal artery, the fluorescence signal clearly revealed the extent of the intrarenal arteriole embolism. In kidneys without embolisms, the fluorescence signal was uniformly distributed over the kidney surface without any defects (Supplementary Fig. S37a and b). However, in embolized kidneys, the fluorescence signal exhibited alternately bright and dark regions on the renal surface (Supplementary Fig. S37c and d). The dark regions represented ischemic areas caused by arteriole embolisms, while the bright areas indicated good kidney perfusion without intrarenal embolisms. For kidneys flushed with larger microspheres, most renal segment arteries were embolized, resulting in the loss of more than two-thirds of the renal surface fluorescence signal (Supplementary Fig. S37e and f). These results suggested the feasibility of NIR-II imaging based on DIPT-ICF NPs for detecting donor kidneys with suboptimal conditions.

**Figure 5.**
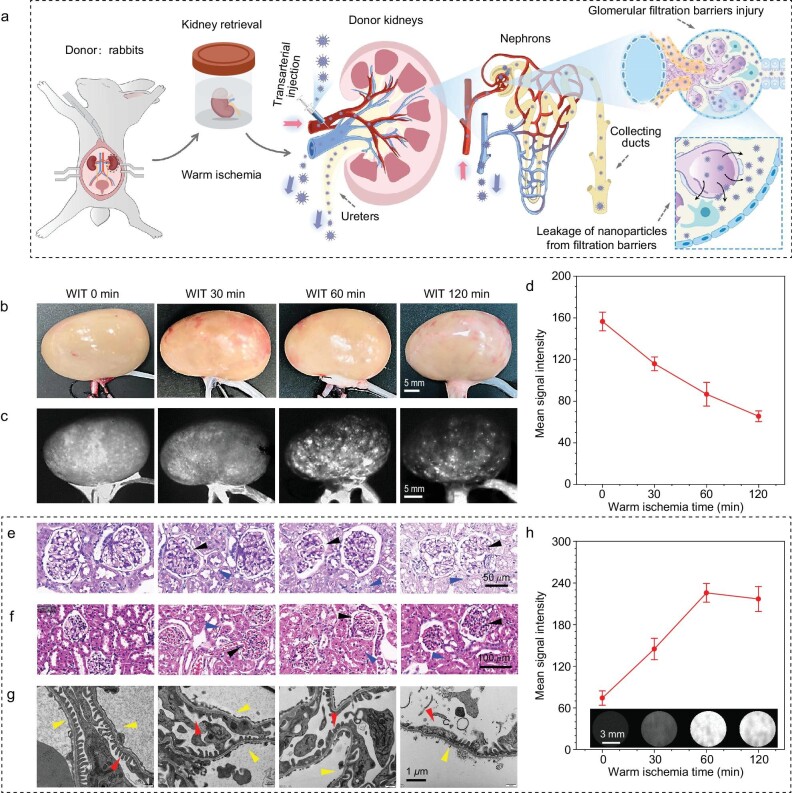
Donor kidney quality assessment prior to implantation using DIPT-ICF NPs as nano-contrast agents. (a) Schematic illustration of warm ischemia-induced GFB injury in donor kidneys during retrieval surgery. DIPT-ICF NPs leak into the urine through damaged GFBs. (b) Color images and (c) NIR-IIa images of the donor kidneys were captured after antegrade injection of DIPT-ICF NPs via the renal artery. (d) Quantitative analysis of NIR-II mean signal intensity of kidneys under different warm ischemia durations in (c). (e) Periodic acid-Schiff (PAS) and (f) hematoxylin-eosin staining of the glomeruli isolated from donor kidneys undergoing different warm ischemia times in (b). With the warm ischemia time increasing, the microvascular network in the glomerulus had degeneration and pyknosis, accompanied by the renal tubular epithelial cells becoming degenerated and necrotic. (g) TEM images of the glomerular filtration barrier, of which the tissue was taken from the donor kidneys undergoing warm ischemia times in (b), respectively. As the warm ischemia time increased, the damage to the three-layer structure of the glomerular basement membrane (GBM) was gradually aggravated, the foot process fusion of podocytes became more and more serious, and the glomerular electron-dense layers got thinner. (h) Quantification of NIR-II mean intensity of the urine excreted from the ureters when the contrast agent of DIPT-ICF NPs was infused into the donor rabbit kidneys via the renal artery. The inset shows NIR-II images of the urine excreted from the ureters during perfusion of DIPT-ICF NPs in donor rabbit kidneys undergoing warm ischemia in (b). The black and blue arrows in (e) and (f) represent atrophied glomeruli and degenerated necrotic tubules due to warm ischemia, respectively; the yellow and red arrows in (g) indicate the endothelial cell disruption and glomerular epithelial cell foot process fusion, respectively. All NIR-II images were captured under a 980-nm laser (60 mW cm^−2^) with a 1319-nm LP filter and an exposure time of 200 ms.

Additionally, donor kidneys may undergo various periods of potential ischemia (Fig. 5a) during kidney transplants despite continuous advances in surgical and hypothermic perfusion techniques [45]. However, as shown in Fig. 5b, the differences in kidneys experiencing varying degrees of warm ischemia could not be identified using the naked eye. With the assistance of NIR-II fluorescence angiography technology, the disparities in kidneys subjected to different ischemia times could be rapidly identified (Fig. 5c). Generally, normal kidneys without ischemic injury exhibited uniform fluorescence throughout the kidney after the injection of DIPT-ICF NPs via the renal artery, whereas the fluorescence signal in injured kidneys transitioned from uniformity to a speckled distribution and decreased with different warm ischemia times (Fig. 5c and d). Periodic acid-Schiff (PAS) staining [46] results indicated that, in warm ischemia donor kidney models, the glomeruli volume decreased, accompanied by enlarged glomerular capsules and vacuolization of the corresponding renal tubular epithelial cells, suggesting the lesioning and necrosis of glomeruli and renal tubules (Fig. 5e). Hematoxylin and eosin (H&E) staining revealed increasing red blood cell trapping and congestion in renal microcirculation with longer ischemia times, leading to the formation of microthrombi and the speckled distribution of fluorescence in the kidney (Fig. 5f). Additionally, DIPT-ICF NPs could not pass through the glomerular filtration barrier (GFB) of normal kidneys (particle size of ∼6 nm makes it difficult to penetrate the GFB) [47], mainly excreting into the gut through bile (Supplementary Fig. S38), appearing in feces (Supplementary Fig. S39) rather than urine (Supplementary Fig. S40). As shown in the TEM images (Fig. 5g), the three layers of the GFB, including the glomerular endothelial cell, glomerular basement membrane (GBM) and podocyte foot processes [48], were damaged during warm ischemia, allowing DIPT-ICF NPs to cross the GFB and enter the urine. Therefore, it is easy to identify GFB damage through urine fluorescence monitoring (Fig. 5h). As an excellent fluorescence contrast agent, DIPT-ICF NPs can be utilized to rapidly assess the reperfusion and function of renal grafts by monitoring NIR-II fluorescence on the kidney surface and in the urine.

## CONCLUSION

In summary, a highly bright NIR-II luminescent nano-contrast agent based on an AIEgen DIPT-ICF has been rationally designed and prepared. This AIE nano-contrast agent, DIPT-ICF NPs, demonstrates superior brightness compared with commercial IR-26 NPs and IR-1061 NPs, outstanding photostability and a prolonged circulation time in comparison with ICG. Moreover, it exhibits excellent biocompatibility, making it an ideal contrast agent for the complex physiological environment of organ transplantation surgeries. These properties collectively position DIPT-ICF NPs as a promising tool for precise and effective organ transplantation surgery. The results indicate that a single dose of DIPT-ICF NPs contrast agent could monitor the entire surgical process very clearly via luminescence due to its high brightness and long-circulating characteristics. This is much better than the traditional luminescent contrast agent of ICG, whose luminescence brightness and circulating time are not sufficient to support the completion of an entire transplantation surgery. In the surgery of living-donor nephrectomy, NIR-II angiography of renal vasculature using DIPT-ICF NPs helps surgeons to visualize the renal vascular branches and their anatomical locations, thereby preventing iatrogenic donor kidney injury. Additionally, real-time observation of surgical complications in vascular and ureterovesical anastomosis is made possible through NIR-II angiography and ureterography utilizing DIPT-ICF NPs as a contrast agent. Furthermore, GFB injury in the donors kidney following warm ischemia and renal graft reperfusion can be evaluated through DIPT-ICF NPs-based NIR-II imaging. The present study showcases an outstanding NIR-II nano-contrast agent and its application in monitoring the entire process of kidney transplantation in experimental animals. It will provide reliable and effective technical insights and support for monitoring and evaluating renal transplantation in the clinic.

## METHODS

### Fabrication of AIE nano-contrast agents

DIPT-IC and DIPT-ICF were synthetized depending on the routes shown in the Supplementary information. Next, a mixture of DIPT-IC or DIPT-ICF (1 mg) and F127 (6 mg) in tetrahydrofuran (THF) solution (5 mL) was poured into deionized water (10 mL). After sonication for 2 min, the suspensions were stirred at room temperature overnight. The mixtures were then dialysed against deionized water for 3 days. After that, the solutions were filtered using 0.22-μm poly (ether sulfone) (PES) membrane filters. The AIE nano-contrast agents were obtained, which were used for further experiments.

### Measurements

The absorption spectra were performed using PerkinElmer Lambda 365 and Shimadzu UV-Vis-NIR Spectrophotometer. PL measurements were performed using a Horiba FluoroMax-4 Spectrofluorometer. Absolute QYs were measured by using a Hamamatsu Quantaurus-QY Plus UV-NIR Instrument. ^1^H and ^13^C spectra were recorded at room temperature on a Unity-400 NMR spectrometer using CDCl_3_ as the solvent and tetramethylsilane as a reference. The size distribution and average size of the DIPT-ICF NPs were performed by DLS using a Zetaplus potential analyser. TEM images were captured by using a FEI Tecnai F20 microscope (accelerating voltage of 200 kV). DFT calculations were carried out by using the B3LYP/6G(d), Gaussian 09 package. All the NIR-II images were captured by using a full-spectrum *in vivo* fluorescence imaging system (Suzhou NIR-Optics Technology Co., Ltd).

## Supplementary Material

nwad286_Supplemental_File
