# Supplementary material for: Bright, photostable and long-circulating NIR-II nanoparticles for whole-process monitoring and evaluation of renal transplantation
Source: Natl Sci Rev. 2023 Nov 8;11(2):nwad286. doi: 10.1093/nsr/nwad286 (PMC10776353; doi:10.1093/nsr/nwad286)
Supplement: nwad286_Supplemental_File [file nwad286_supplemental_file.pdf]

Supplementary Information

Bright, photostable, and long-circulating NIR-II nanoparticles for whole-process monitoring and evaluation of renal transplantation

Rongyuan Zhang[1,2,‡], Ping Shen[3,‡], Yu Xiong[2,‡], Tianjing Wu[3], Gang Wang[3], Yucheng Wang[1], Liping Zhang[1,2], Han Yang[1], Wei He[4,5], Jian Du[6], Xuedong Wei[6], Siwei Zhang[5], Zijie Qiu[1], Weijie Zhang[6,*], Zheng Zhao[1,4,*], Ben Zhong Tang[1,5,7,*]

[1]Clinical Translational Research Center of Aggregation-Induced Emission, The Second Affiliated Hospital, School of Medicine, School of Science and Engineering, Shenzhen Institute of Aggregate Science and Technology, The Chinese University of Hong Kong, Shenzhen (CUHK-Shenzhen), Shenzhen 518172, China;

[2]Center for AIE Research, Shenzhen Key Laboratory of Polymer Science and Technology, Guangdong Research Center for Interfacial Engineering of Functional Materials, College of Materials Science and Engineering, Shenzhen University, Shenzhen 518061, China;

[3]School of Chemistry, Xiangtan University, Xiangtan, 411105, China;
[4]HKUST-Shenzhen Research Institute, Shenzhen 518057, China;
[5]Department of Chemistry, Hong Kong Branch of Chinese National Engineering Research Center for Tissue Restoration and Reconstruction, The Hong Kong University of Science and Technology, Hong Kong, China;

[6]Department of Urology, The First Affiliated Hospital of Soochow University, Suzhou 215006, China;

[7]AIE Institute, Guangzhou Development District, Guangzhou 510530, China

Contents

Experimental Procedures 2
Synthetic Procedures and Characterization Data for the Compounds 6
NMR and Mass Spectra of DIPT-IC and DIPT-ICF 10
Supplementary Figures 18
Supplementary tables 32
Reference 34

Experimental Procedures

Materials

All the chemicals and reagents were purchased from chemical sources, and the solvents for chemical reactions were distilled before use. All air and moisture sensitive reactions were carried out in flame-dried glassware under a nitrogen atmosphere. Pluronic® F-127 and IR-1061 were bought from Sigma-Aldrich. ICG was bought from TCI. IR-26 was obtained from Exciton. Cell Counting Kit-8 (CCK-8) was purchased from DoJindo. Dulbecco's modified Eagle's medium (DMEM), Fetal bovine serum (FBS), penicillin-streptomycin, phosphate-buffered saline (PBS), and trypsin were obtained from ThermoFisher.

Cell Culture.

HeLa, NIH/3T3 and bend.3 cells were cultured in the DMEM containing 10% FBS and 1% penicillin-streptomycin (100 units/mL penicillin and 100 g/mL streptomycin) in a 5% CO_2 humidity incubator at 37 °C.

Cytotoxicity Study of DIPT-ICF NPs.

Dark toxicity: CCK-8 assays were employed to evaluate the cytotoxicity of DIPT-ICF NPs against HeLa cells and NIH/3T3 cells. In brief, HeLa and NIH/3T3 cells were incubated in 96-well plates (Sarsteadt AG & Co. KG., Germany) at a density of 1×10^5 cells/mL. Post 24 h incubation, the cells were exposed to a various of doses of DIPT-ICF NPs. At 24 h post addition of DIPT-ICF NPs, the wells were washed with PBS (1×, pH 7.4) buffer and 100 μL freshly prepared medium containing CCK-8 solution (the volume ratio of CCK-8 was 10%) was added into each well, then the cells were kept in the incubator for 1.5 hours. Cell viability was expressed by the ratio of the absorbance of cells incubated with NPs to that of the cells incubated with culture medium only.

Phototoxicity: NIH/3T3 and bEnd.3 cells were incubated in 96-well plates (Sarsteadt AG & Co. KG., Germany) at a density of 2×10^4 cells/well. After 24 h incubation, the cells were incubated with various concentrations of DIPT-ICF NPs. At 24 h post addition of DIPT-ICF NPs, the wells were washed with PBS (1×, pH 7.4) buffer and

exposed to a 980 nm laser (100 mW cm^{-2}) for 10 min. The incubation was continued for 12 hours, and then 100 μL freshly prepared medium containing CCK-8 solution was added into each well. The cells were kept in the incubator for 1.5 hours. Cell viability was expressed by the ratio of the absorbance of cells incubated with DIPT-ICF NPs to that of the cells incubated with culture medium only.

Animal Experiments.

The mice and New Zealand rabbits were purchased from the Guangdong Medical Laboratory Animal Center. The *in vivo* experiments were carried out with permission from the Guidance Suggestions for the Care and Use of Laboratory Animals. All procedures were approved by the Chinese University of Hong Kong (Shenzhen) (CUHKSZ) and Southern University of Science and Technology (SUSTech) Animal Care and Use Committees.

Kidney Transplantation.

New Zealand rabbits weighing 2.5-3.0 kg were used for establishing orthotopic and ectopic kidney transplantation models.

Anesthesia: Initially, the surgical anesthesia was induced by chloral hydrate (3% W/V) (Merck & Co., Inc.) and Xylazine (3 mg mL^{-1}) (Merck & Co., Inc.) solution through subcutaneous injection. After 15 min, the rabbits were anesthetized with continuous inhaling the mixture of isoflurane (RWD Life Science Co., Ltd.) and oxygen. During anesthesia, normal saline (NS) and glucose solution (5%) were used to maintain blood pressure and replenish energy of rabbit models. The cardiorespiratory monitor (RM500, RWD Life Science Co., Ltd.) was used to monitoring the heart rates, respiratory rates, and oxyhemoglobin saturation of rabbits for real-time adjustment of isoflurane inhalation volume. Using this method, rabbits could safely be held in the anesthetic state for 6-8 hours without mechanical ventilation. Before nephrectomy the model rabbits were injected intravenously with 500 units heparin for systemic heparinization to avoid thrombosis.

Donor kidneys retrieval： An abdominal midline incision (from the xiphoid process to the symphysis) was employed in nephrectomy [3]. Before isolation of the left kidney,

2 mL of hydrochloride lignocaine (0.5%) was injected around the renal vessels to prevent the renal artery spasm produced by dissection. Then the peritoneum over the left kidney, vessels, and ureter was carefully divided, and the renal vessels and ureter were isolated along their full length. 26 G venous catheters were inserted into the proximal end of the renal artery and vein, which were fixed on the vessels. Immediately after the fixation of the catheters, the preservation fluid (4 ^{o}C) was perfused into the left kidney through its artery until the fluid from the left renal vein became clear. Subsequently, the renal artery and the vein were clamped, divided, and ligated, and the kidney removed. The donor kidneys were preserved in cold preservation fluid (4 ^{o}C).

Revascularization: For orthotopic kidney transplantation models, midline incisions were used in the recipient rabbits. Notably, to avoid constriction of mesenteric vasculature and possible development of hypotension and shock, the intestines should keep on the same levels with the edge of the wound. The vascular anastomosis was performed with end-to-end suture. For ectopic kidney transplantation models, lower abdominal midline incisions were adopted in the recipient rabbits. The common iliac artery and common iliac vein were carefully divided. After completely exposing the common iliac vessels, the blood flow of the donor kidneys was reconstructed by means of end-to-side anastomosis. In revascularization processes of orthotopic and ectopic kidney transplantation, the surgery field was magnified by an operating microscope (magnification × 6). The anastomosis was performed with a continuous, atraumatic 8-0 silk suture.

Ureteral reconstruction (Ureteroneocystostomy): Extravesical ureteroneocystostomy was carried out after the donor kidney was perfused with recipient blood and hemostasis has been secured [4]. Firstly, a longitudinal oblique incision was made for approximately 0.5 cm until the bladder mucosa bulges into the incision. Then the bladder mucosa was incised, and 6-0 monofilament absorbable sutures placed through both ends of the incision. The ureter distal end was brought to the mucosa incision, and the mucosal sutures passed through the two sides of the distal end, and the ureter parachuted on to the bladder. The ureter was anastomosed to the bladder mucosa with

continuous sutures between the ureter and the mucosa of the bladder. While the ureteric anastomosis was completed, the seromuscular layer was closed over the ureter with interrupted absorbable sutures.

NIR-II Fluorescence Angiography in Rabbits with Intravenous Injection of DIPT-ICF NPs.

The models of New Zealand rabbits were used for NIR-II fluorescence angiography of the vasculatures with the intravenous injection of DIPT-ICF NPs (0.3 mg mL^{-1}, 1.5 mg kg^{-1}).

Ear angiography: The rabbits were anesthetized with the method described above and fixed properly. Clip the fur from the left rabbit ears and further remove the hair with a depilatory cream. The fluorescence contrast agent DIPT-ICF NPs (0.3 mg mL^{-1}, 1.5 mg kg^{-1}) or ICG (0.05 mg mL^{-1}, 200 μL) was injected through the right ear marginal vein, followed by ear angiography at various time points.

Kidney angiography: The rabbits were anesthetized with the method described above and fixed properly. An abdominal midline incision (from the xiphoid process to the symphysis) was employed to exposure the kidney and its accessory vessels. DIPT-ICF NPs (0.3 mg mL^{-1}, 1.5 mg kg^{-1}) was injected through the ear marginal vein of each rabbit models, then NIR-II imaging were performed.

NIR-II Fluorescence Ureterography in Rabbits with Intraureteral Injection of DIPT-ICF NPs.

After the completion of the ureteroneocystostomy anastomosis, the contrast medium DIPT-ICF NPs (0.1 mg mL^{-1}) was injected antegrade into the ureter through the renal graft pelvis, and NIR-II imaging of the vesicoureteral anastomosis was performed.

Statistical Analysis.

Quantitative data were expressed as mean ± standard deviation. Statistical comparisons were made by Student’s t-test. P value < 0.05 was considered statistically significant.

Synthetic Procedures and Characterization Data for the Compounds

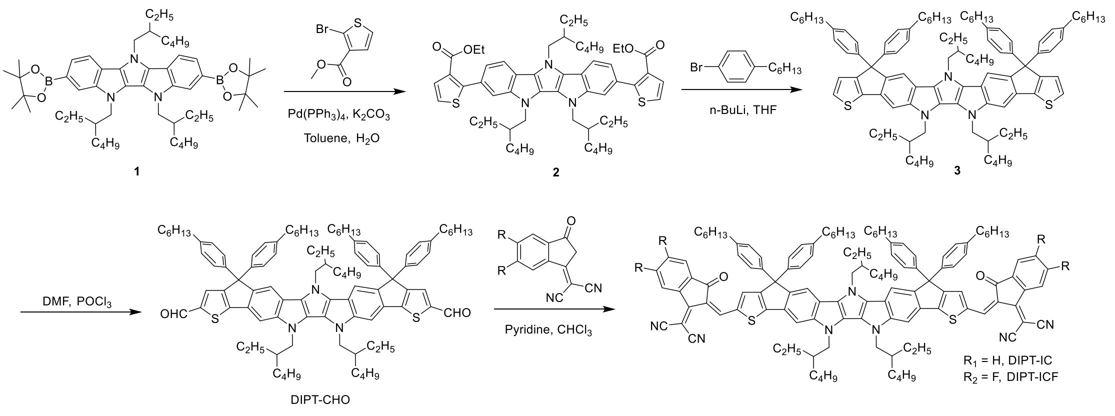

Scheme S1. Synthetic route to DIPT-IC and DIPT-ICF.

Synthetic route of 2.

Compound 1 was synthesized according to our previous literatures [5]. Under argon atmosphere, compound 1 (3.962 g, 4.75 mmol), ethyl 2-bromothiophene-3-carboxylate (3.35 g, 14.25 mmol), Pd (PPh_3)$_4$ (145 mg, 0.12 mmol), K_2CO_3 (1.93 g, 13.96 mmol), distilled water (6 mL), toluene (30 mL), and one drop of trioctylmethylammonium chloride were added to a 100 mL round bottom flask. Slowly heated to 115 °C and stirred for 22 h. After the reaction device was cooled to room temperature, it was extracted with saturated brine and dichloromethane, dried with anhydrous $MgSO_4$ after rotary evaporation, and separated by column chromatography using petroleum ether/dichloromethane as the eluent to obtain compound 2 as a yellow solid (2.75 g, 65% yield). ^{1}H NMR (400 MHz, $CDCl_3$, δ/ppm): 7.74 (d, J = 8.0 Hz, 2H),7.55 (t, J = 4.0 Hz, 4H), 7.61 (s, J = 4.0 Hz, 2H), 7.31 (d, J = 8.0 Hz, 2H), 7.22 (d, J = 8.0 Hz, 2H), 4.49 (t, J = 4.0 Hz, 2H), 4.34 (t, J = 8.0 Hz, 2H), 4.26-4.21 (m, 4H), 2.19 (d, J = 32 Hz, 4H), 1.29-1.19 (m, 26H), 0.90-0.77 (m, 20H); ^{13}C NMR (100 MHz, $CDCl_3$, δ/ppm): 163.67 , 153.06, 139.75, 130.06, 127.33, 125.26, 123.34, 120.32, 119.29, 115.96, 115.87, 111.42, 77.35, 77.03, 76.71, 60.32, 52.02, 50.70, 40.80, 39.86, 30.86, 30.73, 30.68, 29.67, 28.73, 28.59 , 28.50, 24.23, 24.09, 24.06, 22.99, 22.96, 14.10, 13.92, 13.89, 13.87, 10.86, 10.80, 1.00; MS (MALDI-TOF, m/z) $C_{54}H_{71}N_3O_4S_2$: calculated: 890.303; found: 889.859.

Synthetic route of 3.

Under the protection of argon, THF (16 mL) and 1-bromo-4-hexylbenzene (2.85 g, 11.8

mmol) were added to a 100 ml round-bottomed flask at -78 °C, and then 2.4 M n-N Butyllithium in hexane (4.50 mL, 10.82 mmol). After stirring for 2 h at -78 °C, compound 2 (1.751 g, 1.97 mmol) dissolved in THF (15 mL) was slowly added dropwise, kept for 15 min, then moved to room temperature and stirred overnight. After the reaction was completed, quenched with water, extracted with ethyl acetate, and spin-dried, the crude product was transferred to a 250 mL three-necked flask, then acetic acid (40 mL) and octane (40 mL) were added and refluxed for 5 h. After cooling to room temperature, the reaction was quenched with water, extracted with ethyl acetate, and separated by column chromatography using petroleum ether as the eluent to obtain compound 3 as a blue-yellow solid (1.67 g, yield 60%). ^{1}H NMR (400 MHz, Acetone, δ/ppm): 7.73(s, 2H),7.69 (s, 2H), 7.40 (s, 2H), 7.24-7.21 (m, 8H), 7.13 (d, J = 4.0 Hz, 2H), 7.09-7.06 (m, 8H), 4.54-4.47 (m, 4H), 4.40 (t, J = 8.0 Hz, 2H), 2.55 (t, J = 8.0 Hz, 8H), 2.26-2.18 (m, 2H), 1.32 (t, J = 4 Hz, 61H), 0.87-0.83 (m, 20H), 0.77 (t, J = 8.0 Hz,2H), 0.59 (t, J = 8.0 Hz, 4H) ; ^{13}C NMR (100 MHz, Acetone, δ/ppm): 205.30, 155.07, 144.50, 143.77, 143.71, 143.63, 142.12, 140.87, 140.26, 130.54, 128.06, 127.80, 127.78, 127.15, 126.55, 123.40, 119.01, 114.79, 114.23, 101.26, 62.17, 51.14, 50.55, 40.77, 39.96, 35.22, 35.20, 31.54, 31.42, 30.40, 29.52, 29.33, 29.14, 28.95, 28.90, 28.85, 28.76, 28.56, 28.37, 28.29, 24.01, 23.96, 23.66, 22.85, 22.82, 22.72, 22.36, 13.46, 13.34, 13.21, 10.41, 10.37; MS (MALDI-TOF, m/z) $C_{98}H_{127}N_3S_2$: calculated: 1411.241; found: 1410.492.

Synthetic route of DIPT-CHO.

In an argon atmosphere, first add 10 ml of anhydrous DMF to a 100 mL round-bottomed flask, place the reaction device in an ice-water bath to cool to 0 °C, then add 2.03 ml of phosphorus oxychloride dropwise, at 0 °C and after stirring for 2 h, compound 3 (0.721 g, 0.51 mmol) dissolved in 1,2-dichloroethane (10 ml) was slowly added dropwise. After the dropwise addition, the reaction device was moved to an oil bath at 85 °C and stirred for 10 h. After the reaction, the mixture was poured into ice water, stirred for 1 h, extracted with dichloromethane and purified by column chromatography using petroleum ether/dichloromethane (1:1) as eluent to obtain DIPT-CHO (448 mg, 60%

yield). ^{1}H NMR (400 MHz, $CDCl_3$, δ/ppm): 9.81 (s, 2H),7.68 (s, 2H), 7.61 (s, 2H), 7.52 (s, 2H), 7.19 (t, J = 4.0 Hz, 8H), 7.05 (d, J = 8.0 Hz, 8H), 4.41-4.33 (m, 4H), 4.26 (d, J = 8.0 Hz, 2H), 2.54 (t, J = 4.0 Hz, 8H), 2.18-2.06 (m, 2H), 1.97-1.87 (m, 2H), 1.28-1.15 (m, 59H), 0.88-0.84 (m, 20H), 0.70 (t, J = 8.0 Hz,4H), 0.58 (t, J = 8.0 Hz,4H); ^{13}C NMR (100 MHz, $CDCl_3$, δ/ppm): 180.35,142.88, 142.00, 128.81, 128.11, 77.79, 77.68, 77.48, 77.16, 62.74, 5, 32.16, 5, 8 , 23.05, 14.54, 14.43, 1.48, 0.46; MS (MALDI-TOF, m/z) $C_{100}H_{127}N_3O_2S_2$: calculated: 1467.260; found: 1465.850.

Synthesis of small molecule DIPT-IC.

Under argon atmosphere, DIPT-CHO (0.42 g, 0.28 mmol), 2-(3-oxo-2,3-dihydro-1H-inden-1-ylidene) malononitrile (IC) (0.22 g, 1.12 mmol), pyridine (1 mL) and chloroform (40 mL) were sequentially added into a 100 mL round bottom flask and refluxed for 8 h. After the reaction, the mixture was dropped into methanol (200 mL) to settle, left to stand, filtered with suction, and the solid was collected. Column chromatography using petroleum ether/dichloromethane as the eluent gave DIPT-IC as a bright black solid (0.36 g, yield 70%). ^{1}H NMR (400 MHz, $CDCl_3$, δ/ppm) δ 8.87 (s, 2H), 8.65 (s, 2H), 7.89 (s, 2H), 7.68 (d, J = 12 Hz 8H), 7.60 (s, 2H), 7.19 (d, J = 4 Hz,8H), 7.07 (d, J = 8 Hz,8H), 4.40-4.28 (m, 4H), 4.3 (d, J = 8 Hz, 2H), 2.56 (t , J = 8 Hz, 8H), 2.22-2.10 (m, 2H), 1.97-1.86 (m, 2H), 1.42-1.15 (m, 59H), 0.92-0.82 (m, 20H), 0.71 (t, J = 8 Hz,4H), 0.60 (t, J = 8 Hz, 4H); ^{13}C NMR (100 MHz, $CDCl_3$, δ/ppm): ^{13}C NMR (101 MHz, $CDCl_3$) δ 188.85, 163.47, 160.76, 157.37, 147.30 , 142.13, 141.92, 141.46, 140.18, 140.10, 138.35, 136.99, 134.87, 134.14, 129.87, 129.57, 128.63, 127.83, 125.23, 123.47, 121.10, 120.23, 117.10, 115.33, 115.05, 104.05, 77.48, 77.16, 76.84, 67.39 , 62.33, 51.28, 40.04, 35.68, 31.84, 31.51, 30.87, 30.71, 29.25, 29.22, 28.78, 28.61, 24.30, 23.91, 23.21, 22.98, 22.72, 14.21, 14.07, 13.81, 11.08, 11.02, 10.74, 1.16; MS (MALDI-TOF, m/z) $C_{124}H_{135}N_7O_2S_2$: calculated: 1819.611; found: 1818.794.

Synthesis of small molecule DIPT-ICF.

Similar to the synthesis method of DIPT-IC, DIPT-ICF was finally obtained as a blue-black solid (0.35 g, yield 63%) ^{1}H NMR (400 MHz, $CDCl_3$, δ/ppm) δ 8.87 (s, 2H),

8.54-8.50 (m, 2H), 7.71-7.63 (m, 6H), 7.16 (t, J = 4 Hz 8H), 7.07 (d, 4 Hz ,8H), 4.40-4.27 (m, 4H), 4.24(d, J = 4 Hz, 2H), 2.20-2.09 (m, 2H), 1.95-1.86 (m, 2H), 1.28-1.15 (m, 59H), 0.92-0.88 (m, 20H), 0.85 (t, J = 8 Hz 4H), 0.60 (t, J = 8 Hz 4H); ^{13}C NMR (100 MHz, $CDCl_3$, δ/ppm): 186.46, 164.63, 157.78, 147.54, 142.03, 141.57, 140.05, 138.36, 130.148, 6 ; MS (MALDI-TOF, m/z) $C_{124}H_{131}F_4N_7O_2S_2$: calculated: 1819.571; found: 1819.150.

Fabrication of DIPT-IC NPs and DIPT-ICF NPs.

A mixture of DIPT-IC or DIPT-ICF (1 mg) and Pluronic® F-127 (4 mg) in tetrahydrofuran (THF) solution (2 mL) was slowly dropped into deionized water (Milli-Q, France) (10 mL). The mixtures were sonicated for 5 min (microtip probe sonicator at 50 W output, Scientz-IID, Ningbo Scientz Biotechnology Co., Ltd.) in an ice bath, then which were stirred vigorously at room temperature overnight. The mixtures were dialyzed against deionized water for 48 hours and then concentrated to 0.5 mg mL^{-1} using ultrafiltration centrifuge tubes (100 KD, Merck Millipore, Ireland). After that, the suspensions were filtered using a 0.22 μm Millipore Express poly (ether sulfone) (PES) membrane filter (Merck Millipore, Ireland). The clear suspension of DIPT-IC NPs and DIPT-ICF NPs were obtained, which were used for further experiments.

NMR and Mass Spectra of DIPT-IC and DIPT-ICF

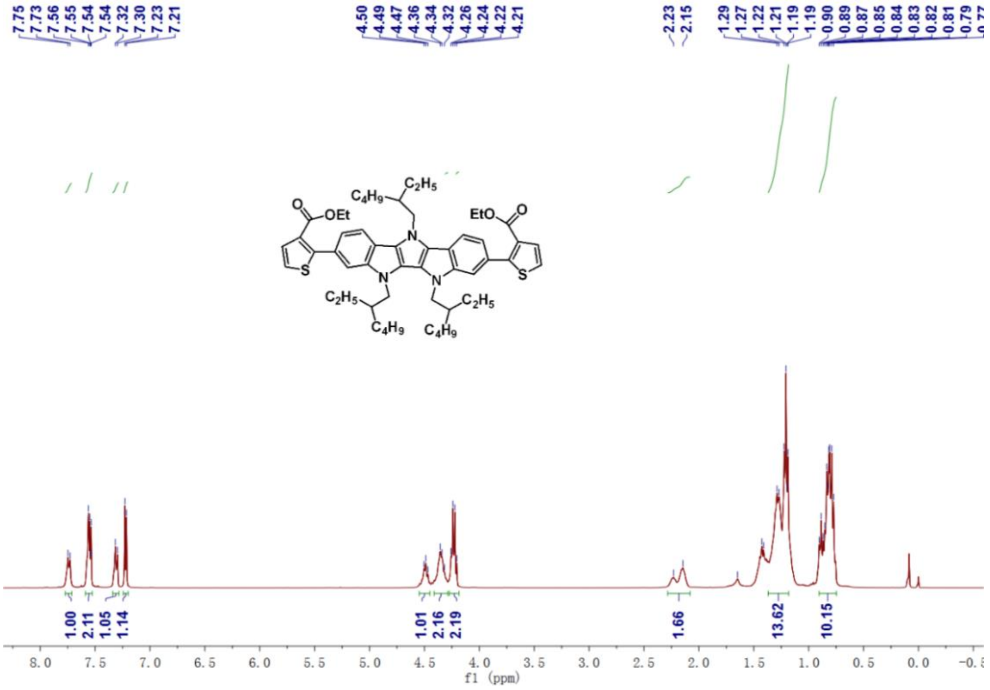

Figure S1. ^{1}H NMR spectrum of compound 2.

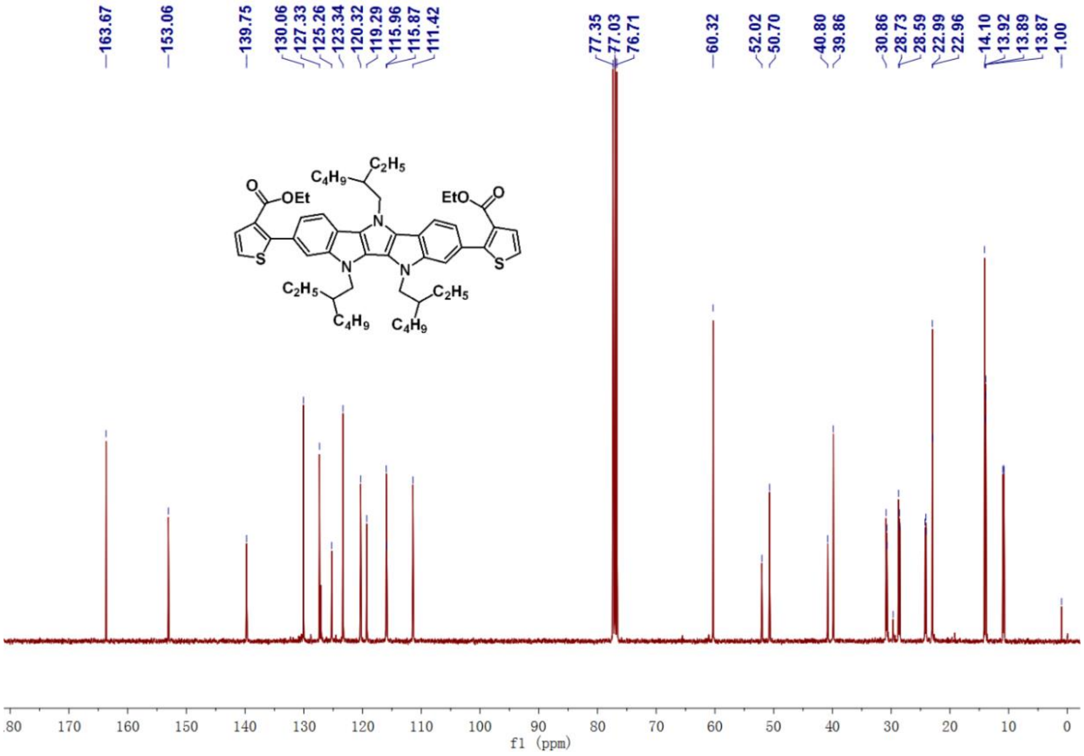

Figure S2. ^{13}C NMR spectrum of compound 2.

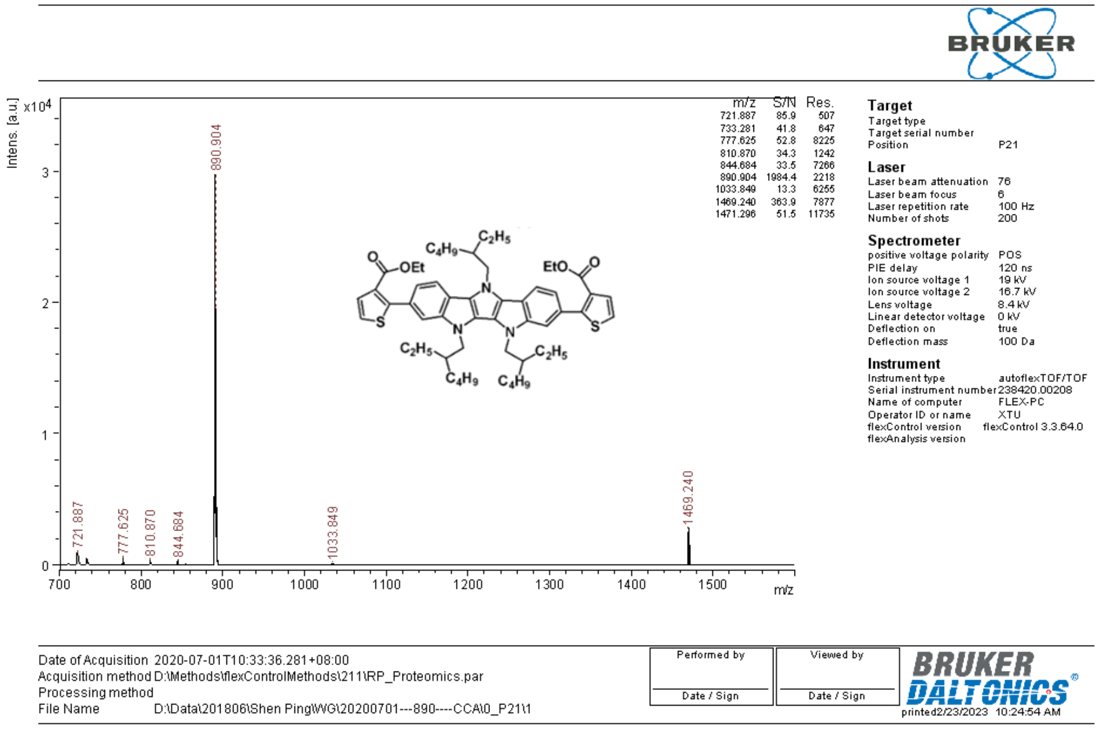

Figure S3. MALDI-TOF MS spectrum of compound 2.

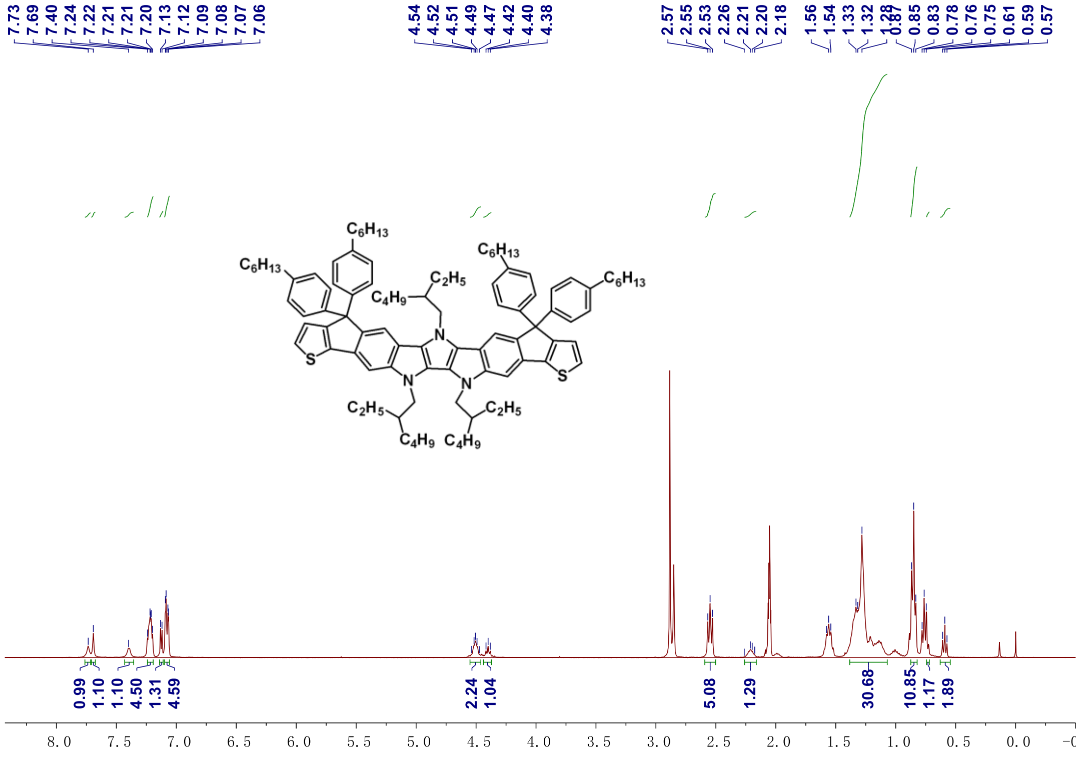

Figure S4. ^{1}H NMR spectrum of compound 3.

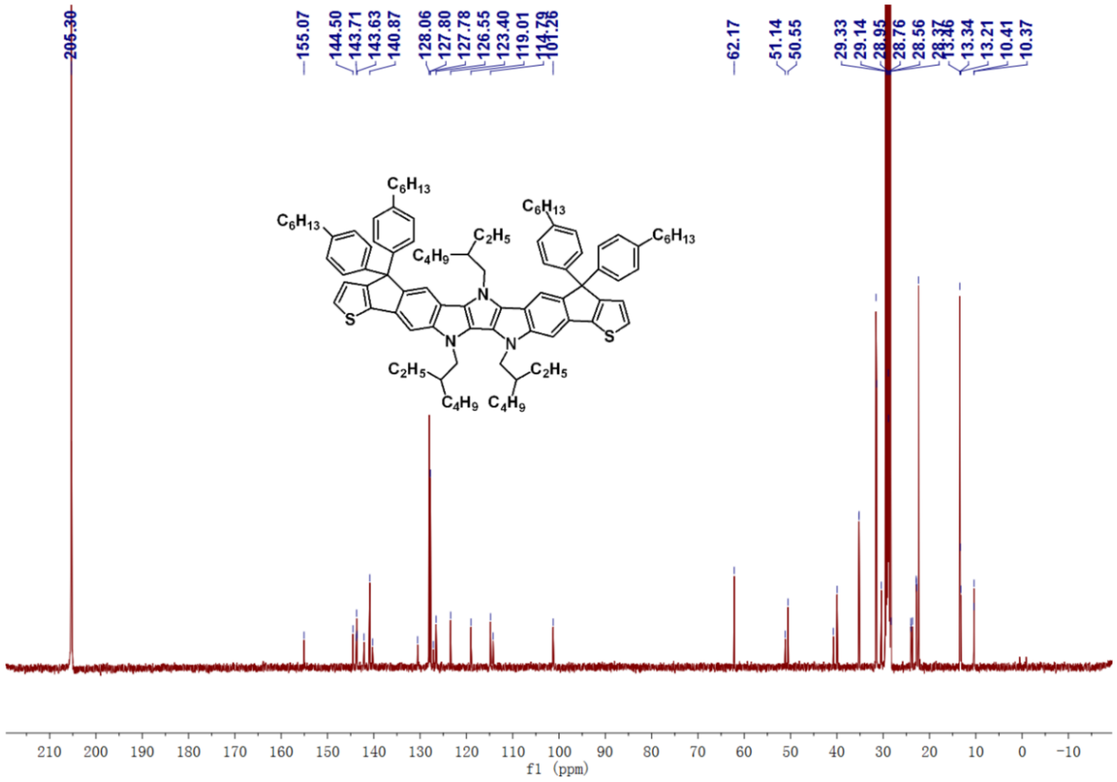

Figure S5. ^{13}C NMR spectrum of compound 3.

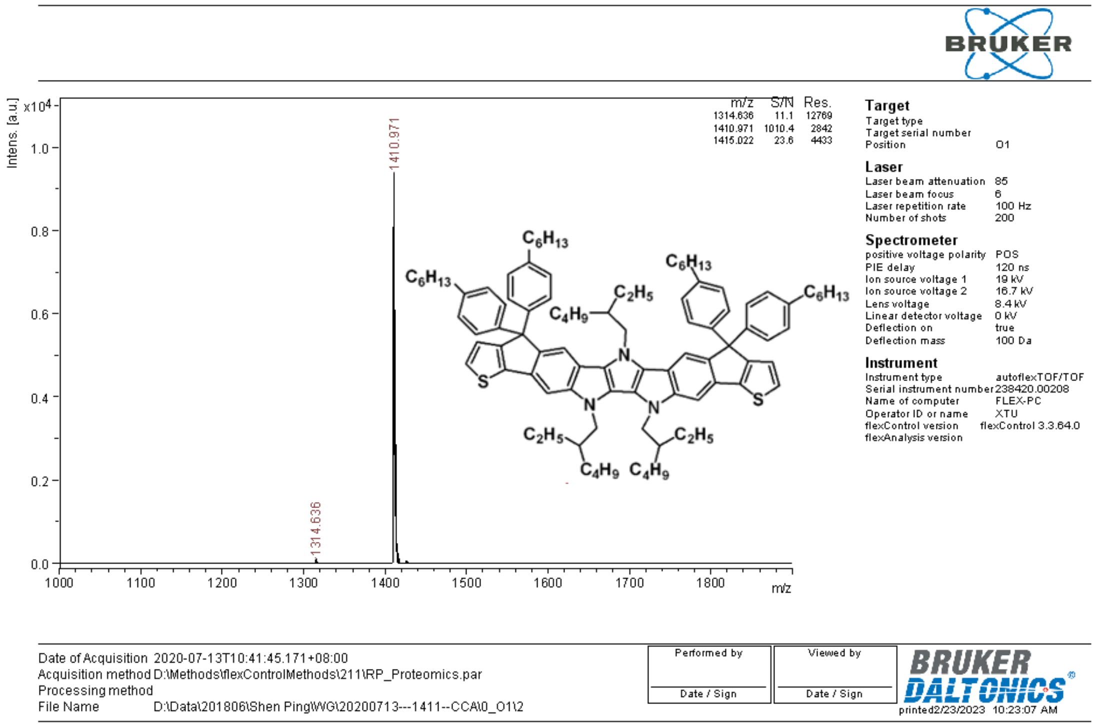

Figure S6. MALDI-TOF MS spectrum of compound 3

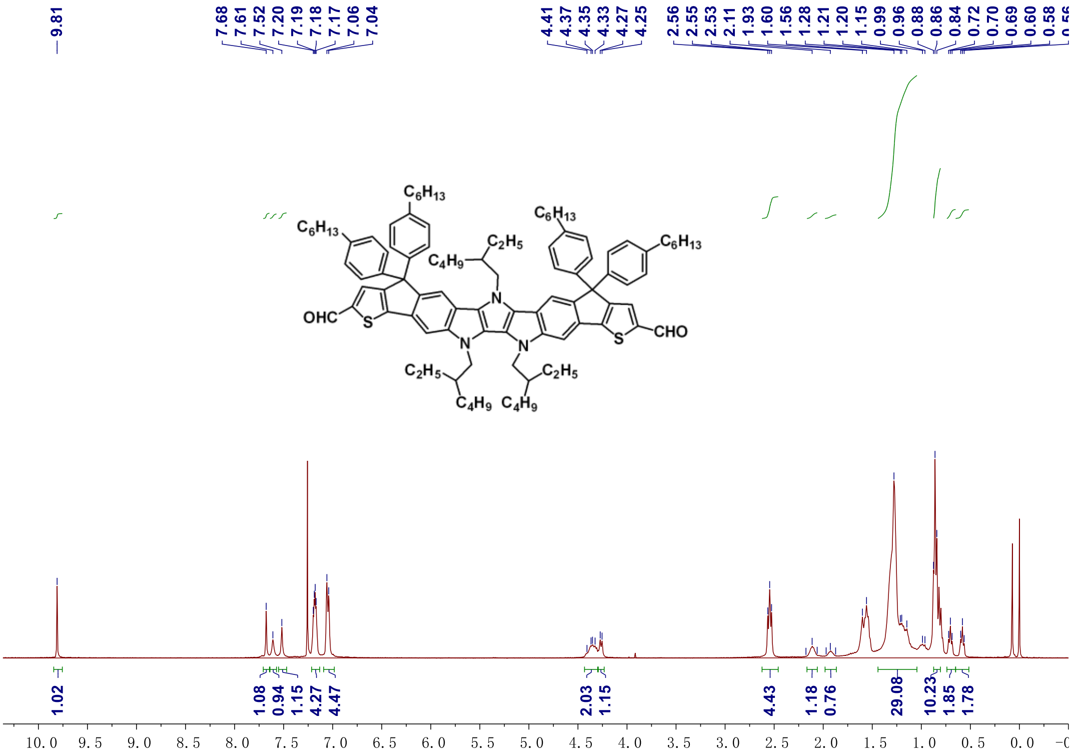

Figure S7.^{1}H NMR spectrum of DIPT-CHO

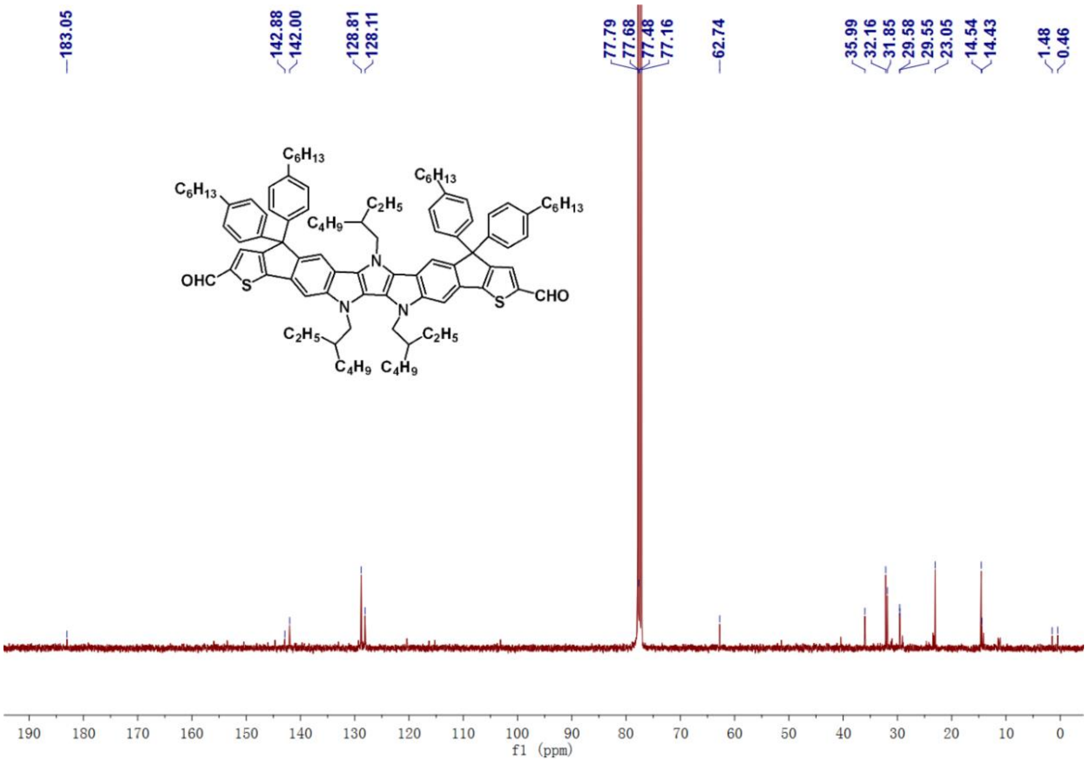

Figure S8. ^{13}C NMR spectrum of DIPT-CHO

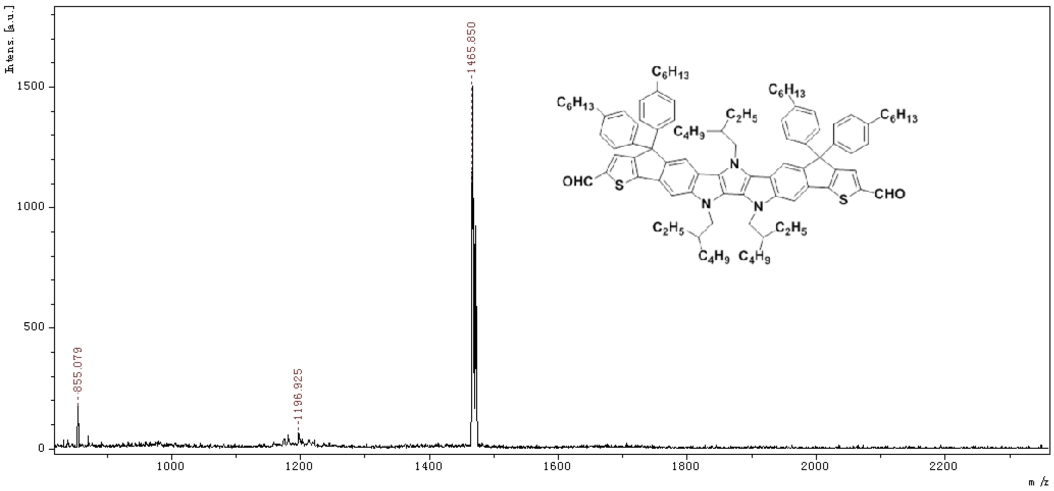

Figure S9. MALDI-TOF MS spectrum of DIPT-CHO

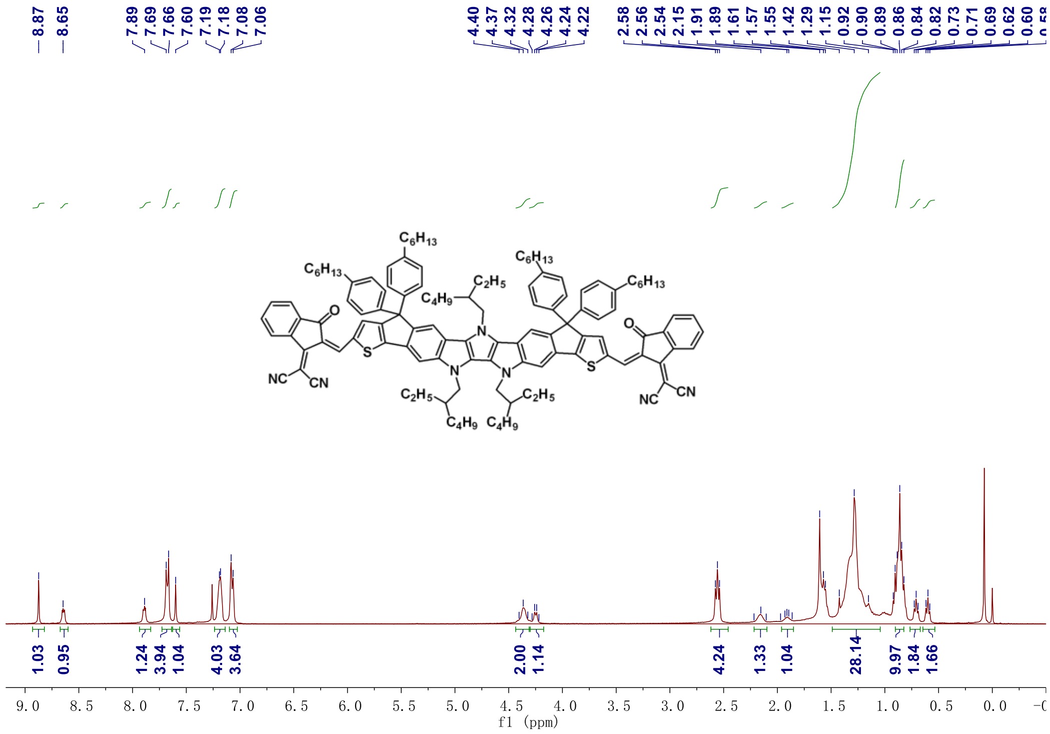

Figure S10. ^{1}H NMR spectrum of DIPT-IC

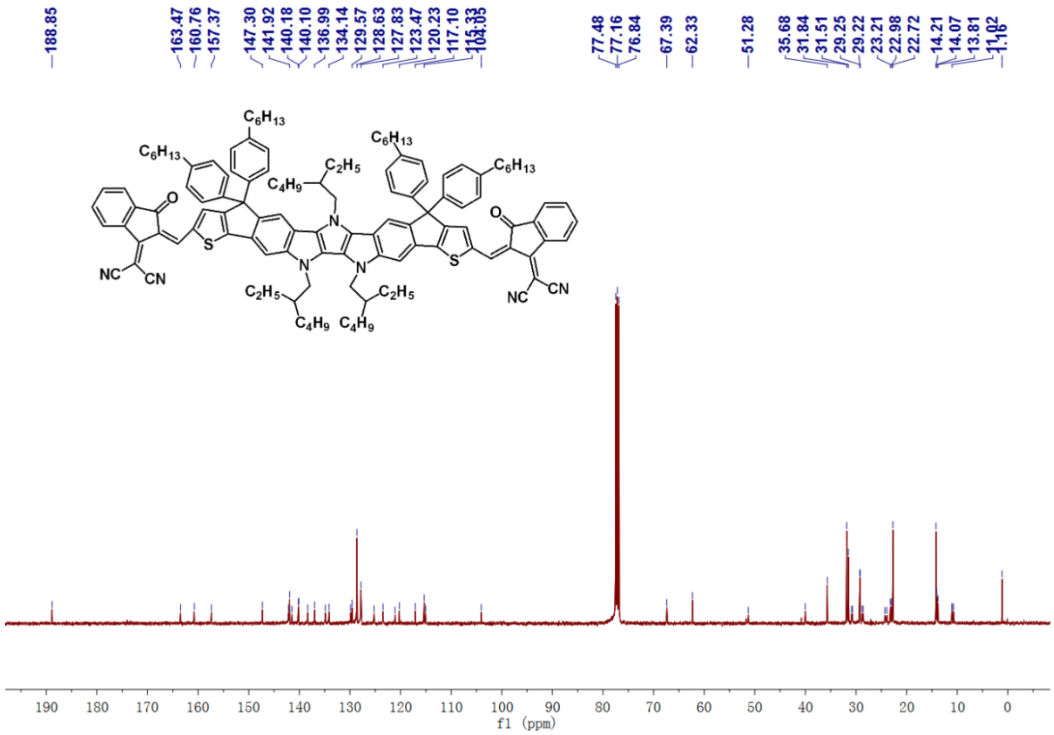

Figure S11. ^{13}C NMR spectrum of DIPT-IC

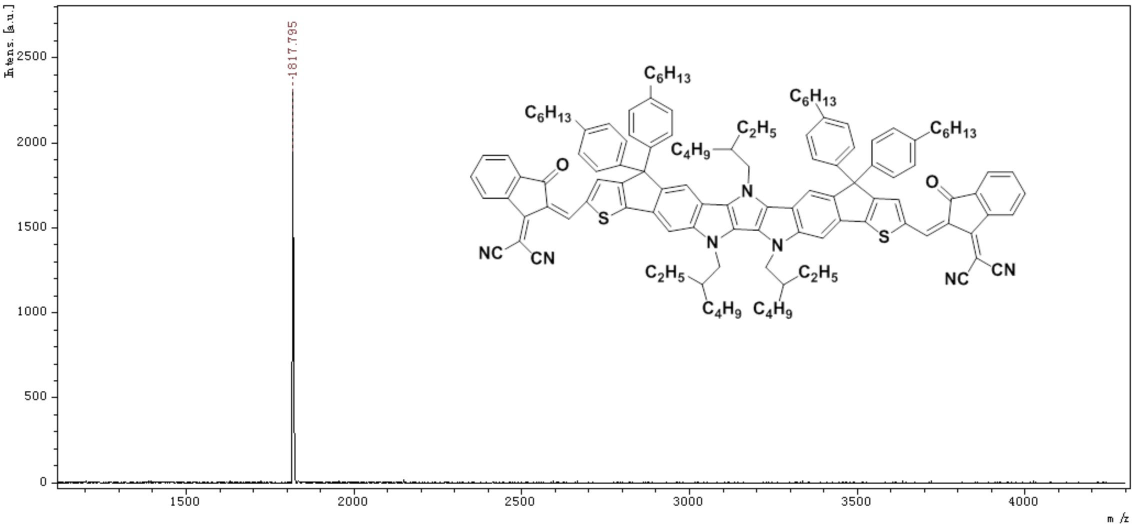

Figure S12. MALDI-TOF MS spectrum of DIPT-IC

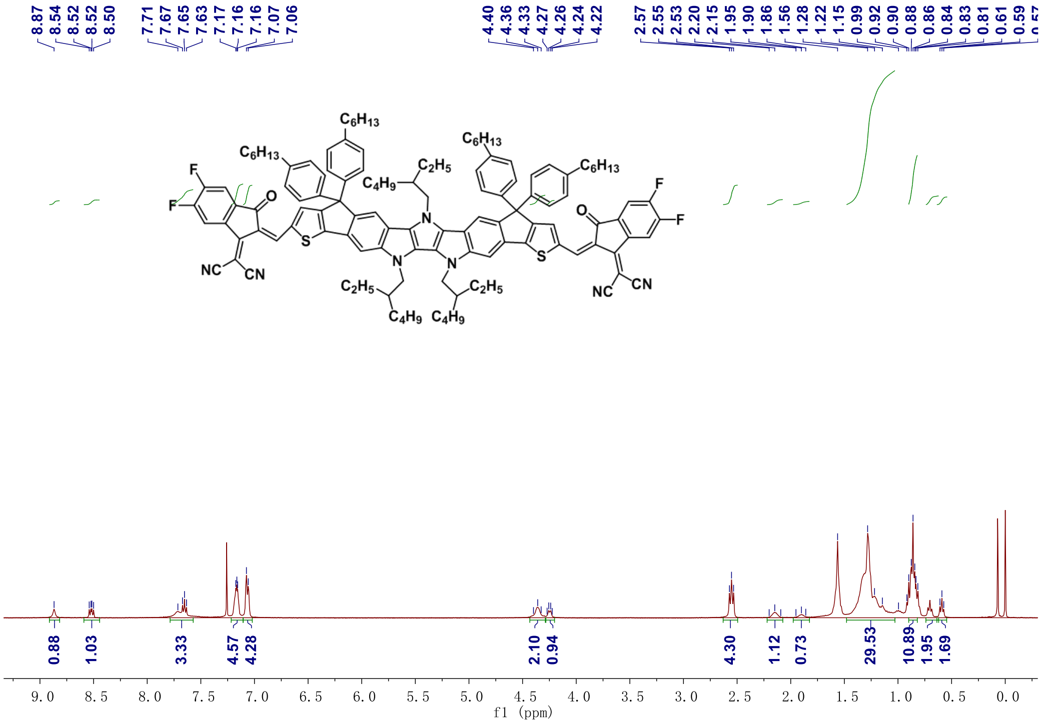

Figure S13. ^{1}H NMR spectrum of DIPT-ICF

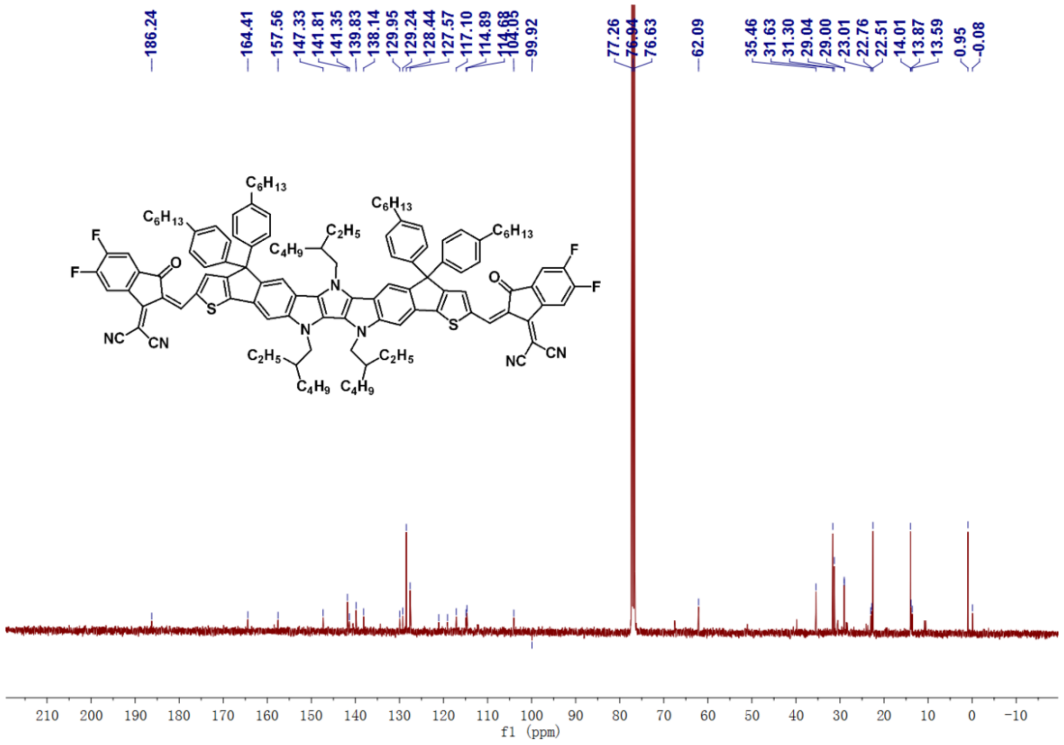

Figure S14. ^{13}C NMR spectrum of DIPT-ICF

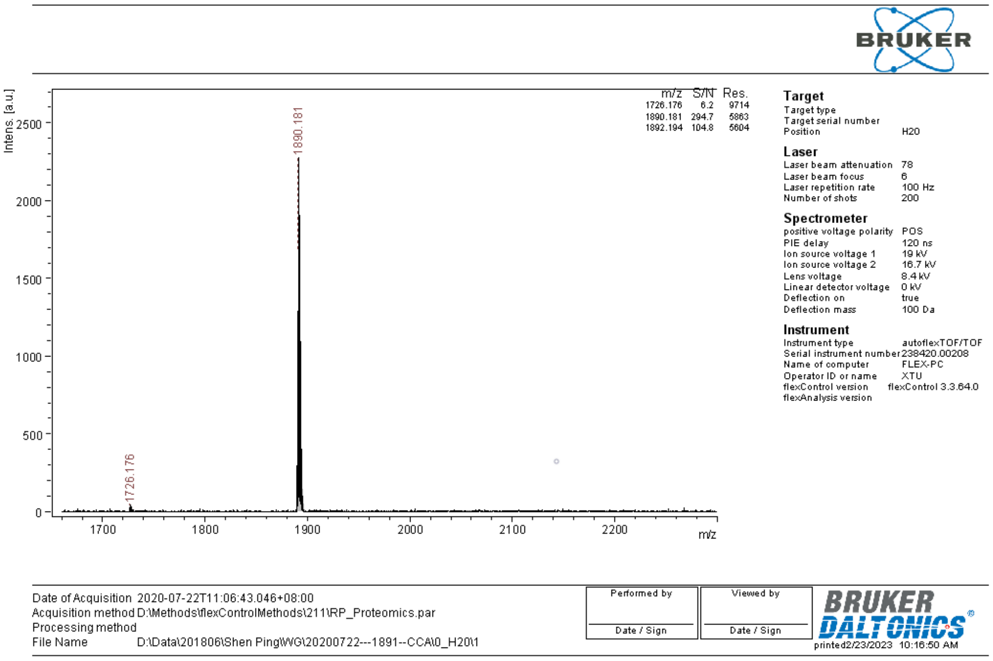

Figure S15. MALDI-TOF MS spectrum of DIPT-ICF

Supplementary Figures

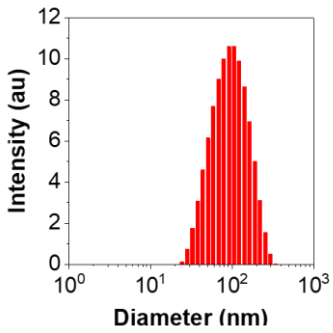

Figure S16. DLS data of DIPT-IC NPs.

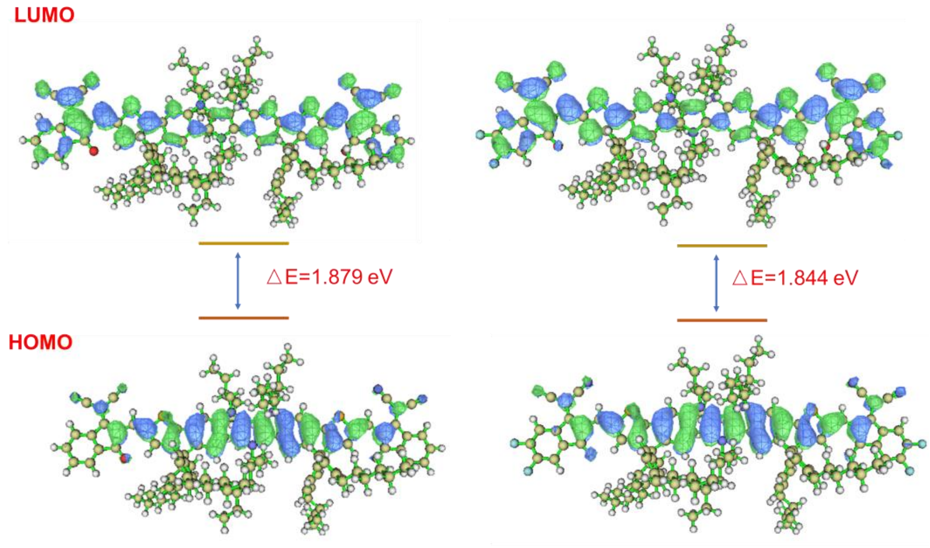

Figure S17. The spatial plots of HOMO and LUMO at ground-state geometry (iso-value = 0.03) based on B3LYP/6-31g (d, p) level.

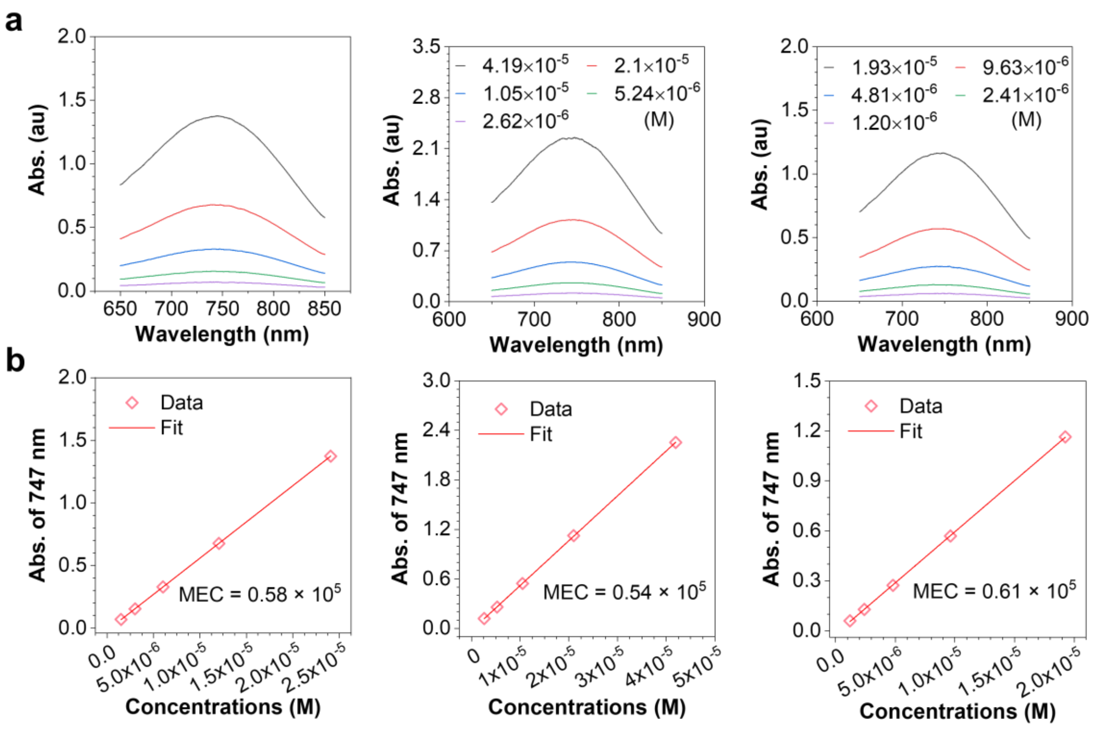

Figure S18. MEC calculation of DIPT-IC. (a) Absorption spectra of DIPT-IC solution (solvent THF) at different molar concentrations (n = 3, three independent replicate measurements). (b) The absorbance at 747 nm of DIPT-IC solution in different molar concentrations (corresponding to panel a) and their fitted curves, with slopes representing the MEC. The ε of DIPT-IC in the THF solution can be calculated as 0.58 $\pm 0.035 \times 10^5$ $M^{-1}cm^{-1}$ from these three independent replicate measurements.

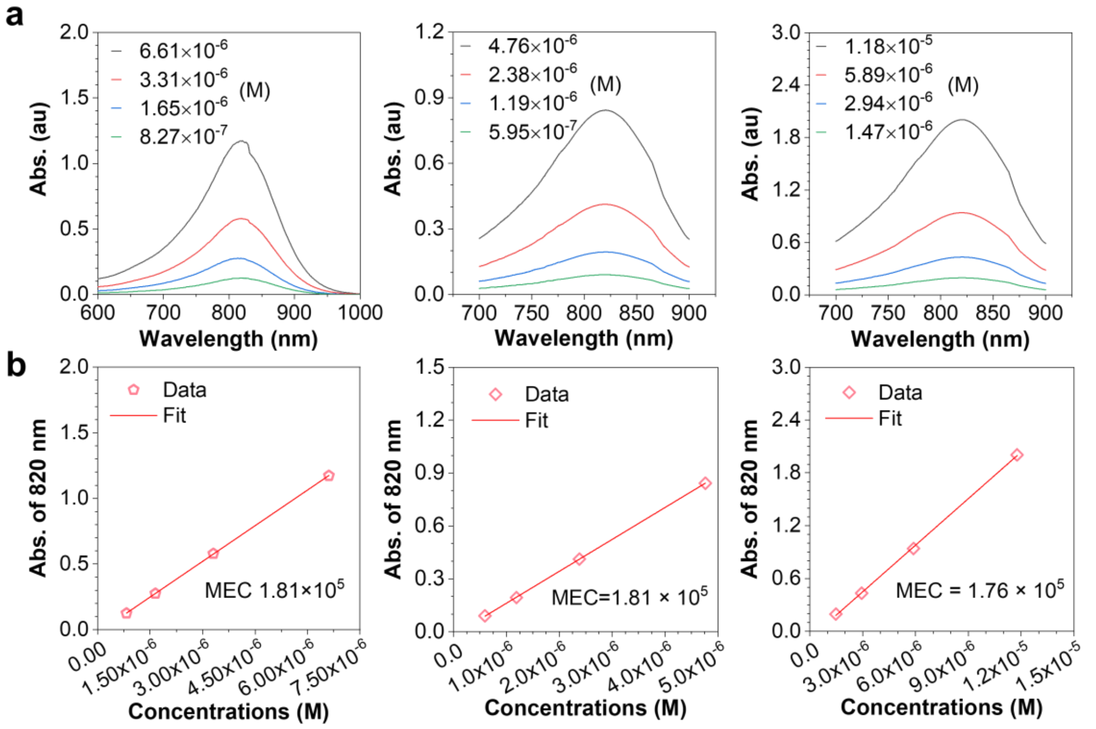

Figure S19. MEC calculation of DIPT-ICF. (a) Absorption spectra of DIPT-ICF

solution (solvent THF) at different molar concentrations (n = 3, three independent replicate measurements). (b) The absorbance at 820 nm of DIPT-ICF solution in different molar concentrations (corresponding to panel a) and their fitted curves, with slopes representing the MEC. The ε of DIPT-ICF in the THF solution can be calculated as $1.79 \pm 0.03 \times 10^5$ $M^{-1}cm^{-1}$ from these three independent replicate measurements.

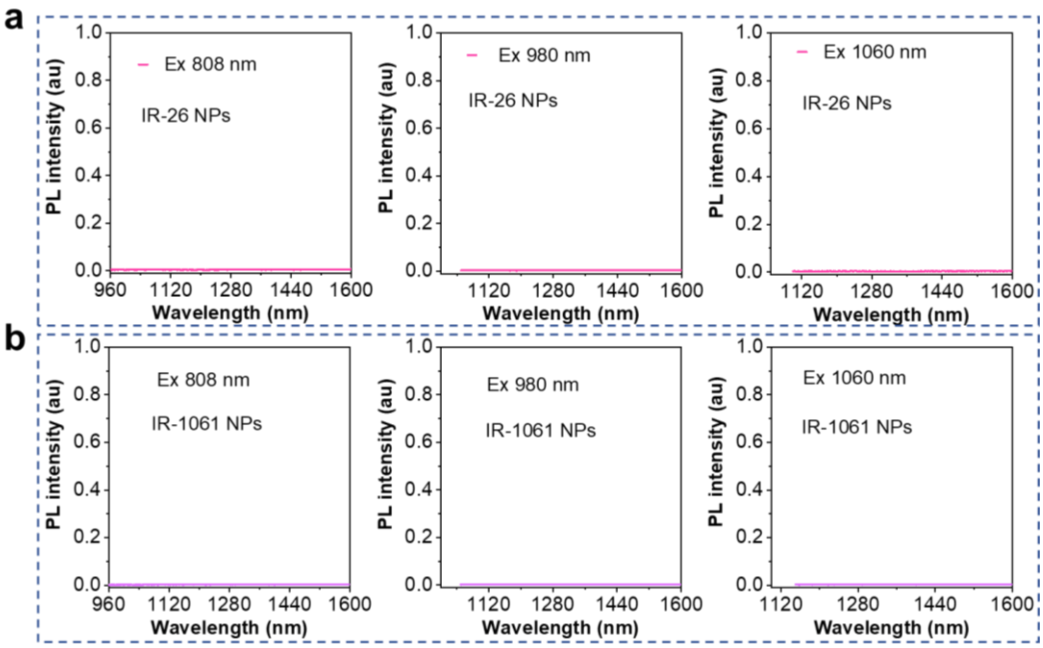

Figure S20. Emission spectra of (a) IR-26 NPs and (b) IR-1061 NPs dispersed in DI water. The excitation sources are 808, 980, and 1060 nm.

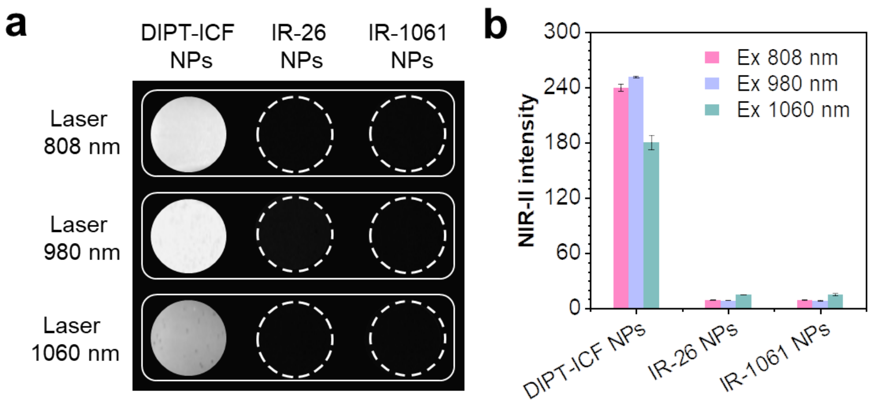

Figure S21. NIR-IIa imaging effect assessments of DIPT-ICF-/ IR-26-/ IR-1061 NPs dispersed in DI water *in vitro*. (a) NIR-IIa images captured from an *in vivo* imaging system, using 808 nm, 980 nm, and 1060 nm lasers as excitation sources. (b) Quantitative analysis of NIR-IIa signal intensity in panel (a).

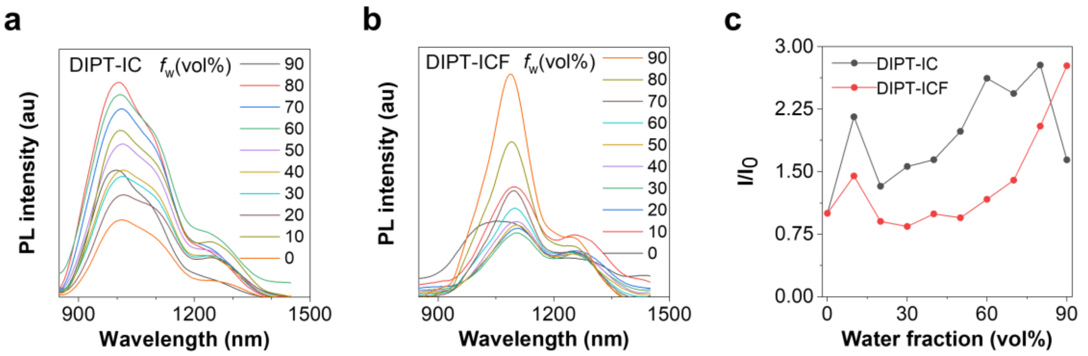

Figure S22. PL spectra of DIPT-IC (a) and DIPT-ICF (b) in a mixture of DMF/water with different f_w values. (c) PL intensity variation with water fraction in DMF/H_2O mixtures.

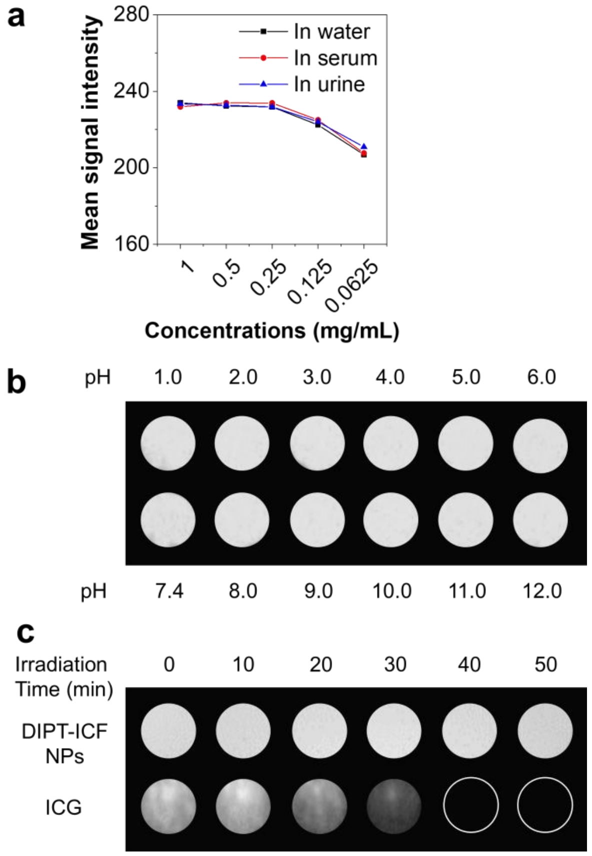

Figure S23. (a)The quantitative analysis of the signal intensity in Figure 1f. (b) NIR-II images of DIPT-ICF NPs dispersion with different pH value. (c) Photostability evaluation of the DIPT-ICF NPs dispersion and ICG solutions upon constant laser beam exposure for various times.

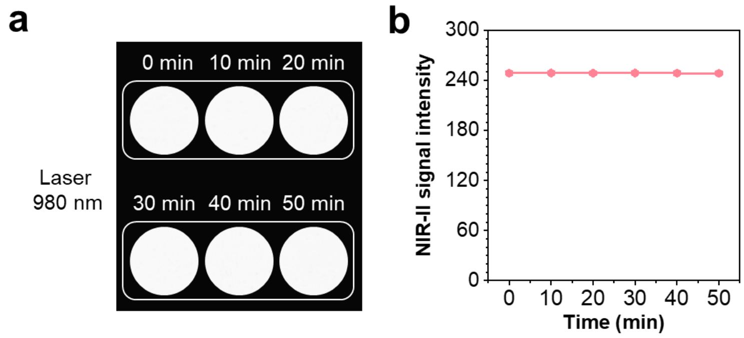

Figure S24. Photostability evaluation of DIPT-ICF NPs upon 980 nm irradiation for 50 min.

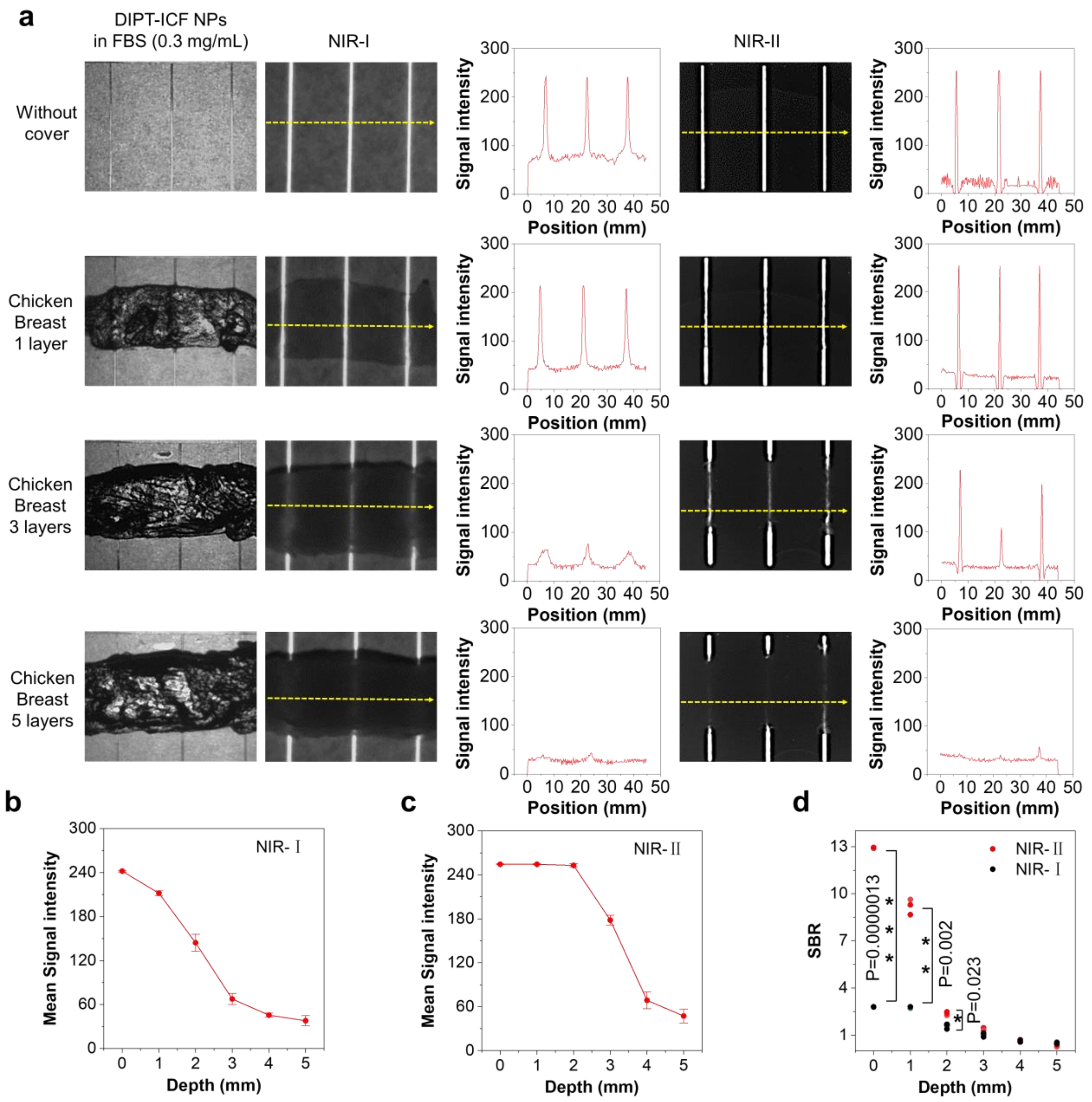

Figure S25. Comparison of the penetration and resolution of NIR-II and NIR-I imaging. (a) Images of the capillary glass tubes containing DIPT-ICF Aggs dispersed in FBS. Yellow arrows correspond to the location and direction of cross-sectional fluorescence intensity profiles for both the NIR-I and NIR-II regions. The capillary glass tubes were

covered by chicken breast tissue of various thicknesses (1.0, 3.0, and 5.0 mm). (b, c) The NIR-I/II fluorescence signal intensity decreased with increasing depth of the capillary glass tubes. (d) There was a significant difference between the signal-background-ratio (SBR) of NIR-II and NIR-I imaging at various depths within 3 mm ($P < 0.05$). Significance was calculated by a Student's t-test. ***$P < 0.001$, **$P < 0.01$, *$P < 0.05$.

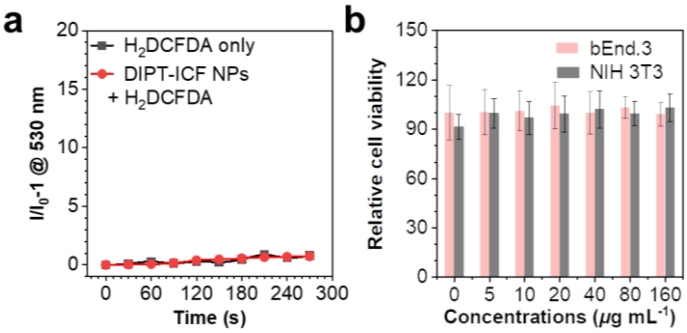

Figure S26. Assessments of ROS generation and phototoxicity of DIPT-ICF NPs upon a 980 nm laser irradiation (100 mW cm^{-2}). (a) ROS generation assessment of DIPT-ICF NPs (0.1 mg mL^{-1}) using H2DCFDA (4×10^{-5} M) as an indicator. (b) Phototoxicity evaluation of cells bEnd.3 and NIH/3T3 by CCK-8 assay.

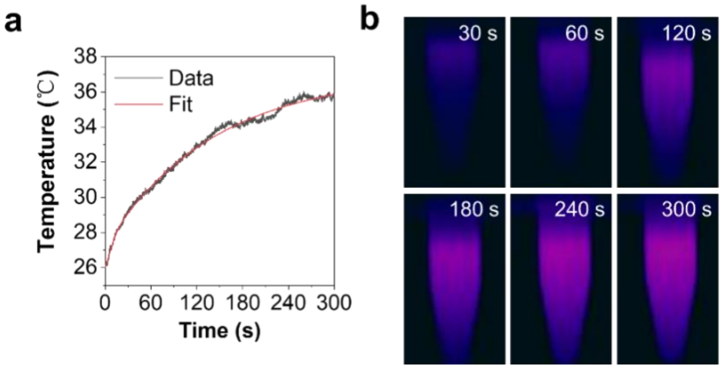

Figure S27. Photothermal characterization of DIPT-ICF NPs. (a) The temperature monitoring curves of DIPT-ICF NPs (0.05 mg mL^{-1}), dispersed in PBS under the continuous laser irradiation for 5 minutes (980 nm, 100 mW cm^{-2}). (b) The infrared thermographic images of DIPT-ICF NPs were captured at several time points (30, 60, 120, 180, 240, and 300 s) during 980 nm laser irradiation.

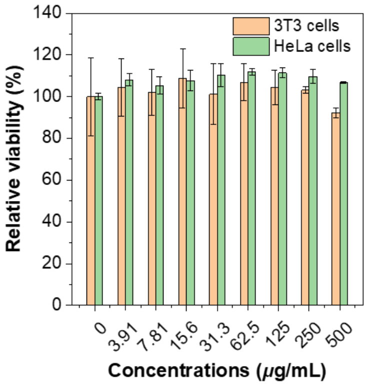

Figure S28. Dark toxicity assessment of 3T3 and HeLa cells after incubation with DIPT-ICF NPs at various concentrations for 24 h.

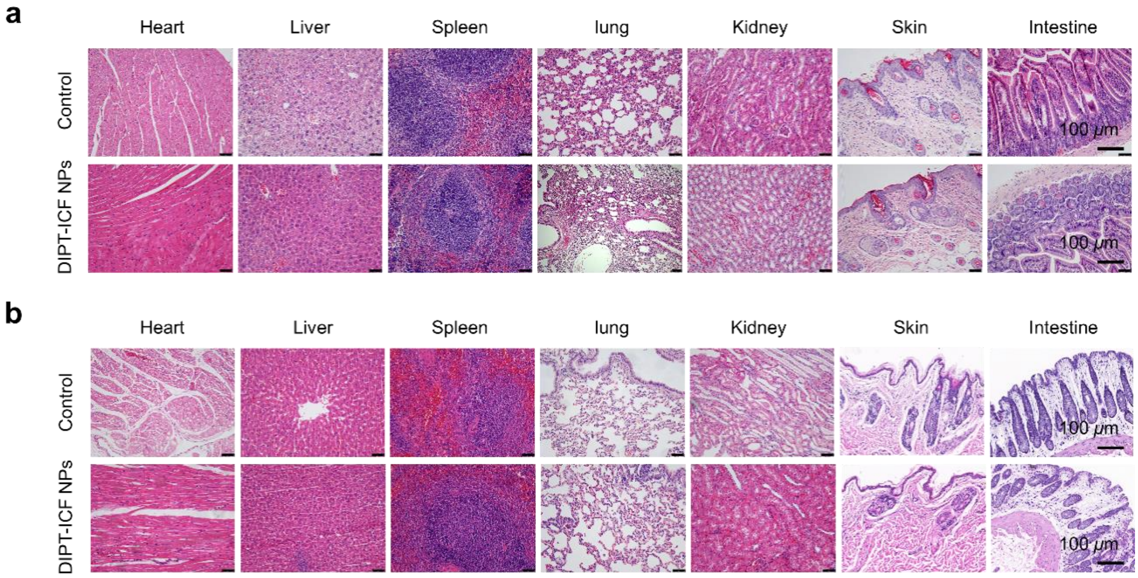

Figure S29. Histological H&E staining for hearts, livers, spleens, lungs, kidneys, skin, and intestines of rabbits **(a)** and mice **(b)** on day 14 after DIPT-ICF NPs treated. The untreated rabbits and mice were used as the control.

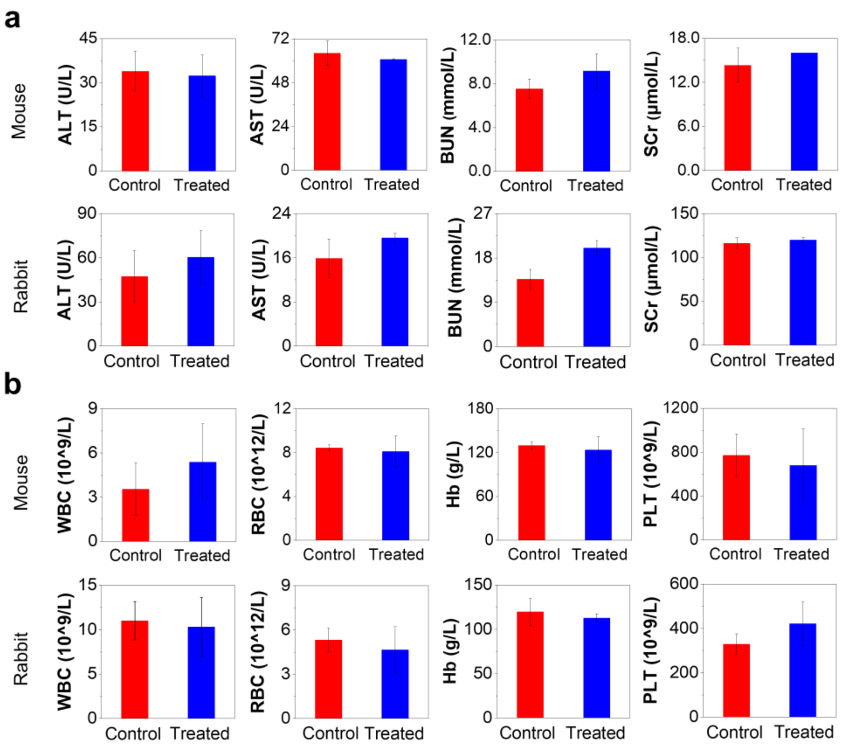

Figure S30. Blood test parameters in terms of (a) liver and renal function as well as (b) white blood cell, red blood cell, haem regulation, and platelets count of healthy Balb/c nude mice and rabbits intravenously injected with DIPT-ICF NPs for 14 days. The untreated mice and rabbits were used as the control.

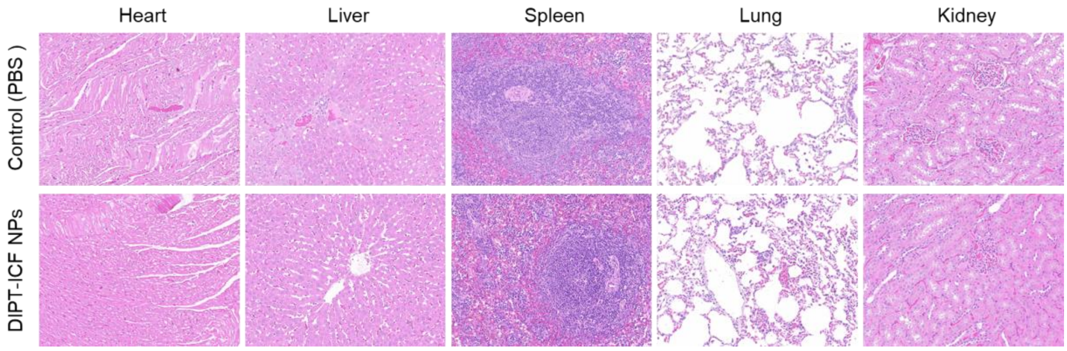

Figure S31. Histological H&E staining had been performed on the main organs (hearts, livers, spleens, lungs, and kidneys) of a rabbit after injection of PBS and DIPT-ICF NPs for 43 days.

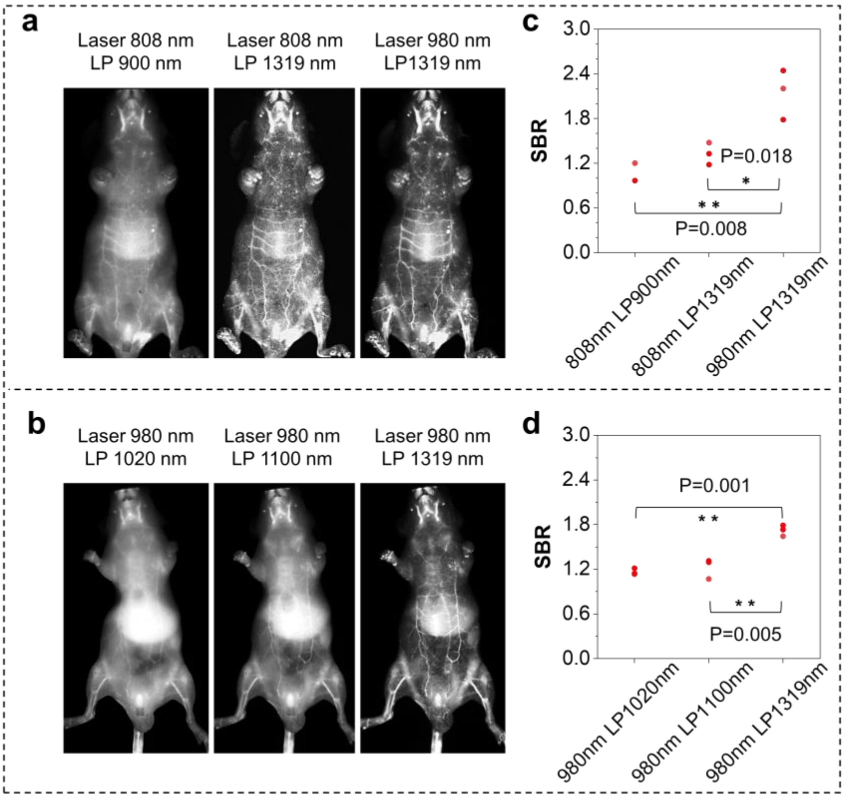

Figure S32. NIR-I/II subcutaneous angiography with DIPT-ICF NPs in mice. (a) NIR-II subcutaneous angiography was performed on mice abdomens under various lasers and filters arrays, such as 808 nm laser with 900 nm long pass filter (LP 900 nm), 808 nm laser with 1319 nm long pass filter (LP 1319 nm), and 980 nm laser with LP 1319 nm. (b) NIR-II subcutaneous angiography was performed on the abdomen of mice under a 980 nm laser with LP 1020 nm, LP 1100 nm, and LP 1319 nm. (c, d) Quantitative analysis indicated that the SBR of angiography under the parameter of 980 nm laser with LP 1319 nm was highest among all the arrays. Significance was calculated by a Student's t-test. **$P < 0.01$, *$P < 0.05$.

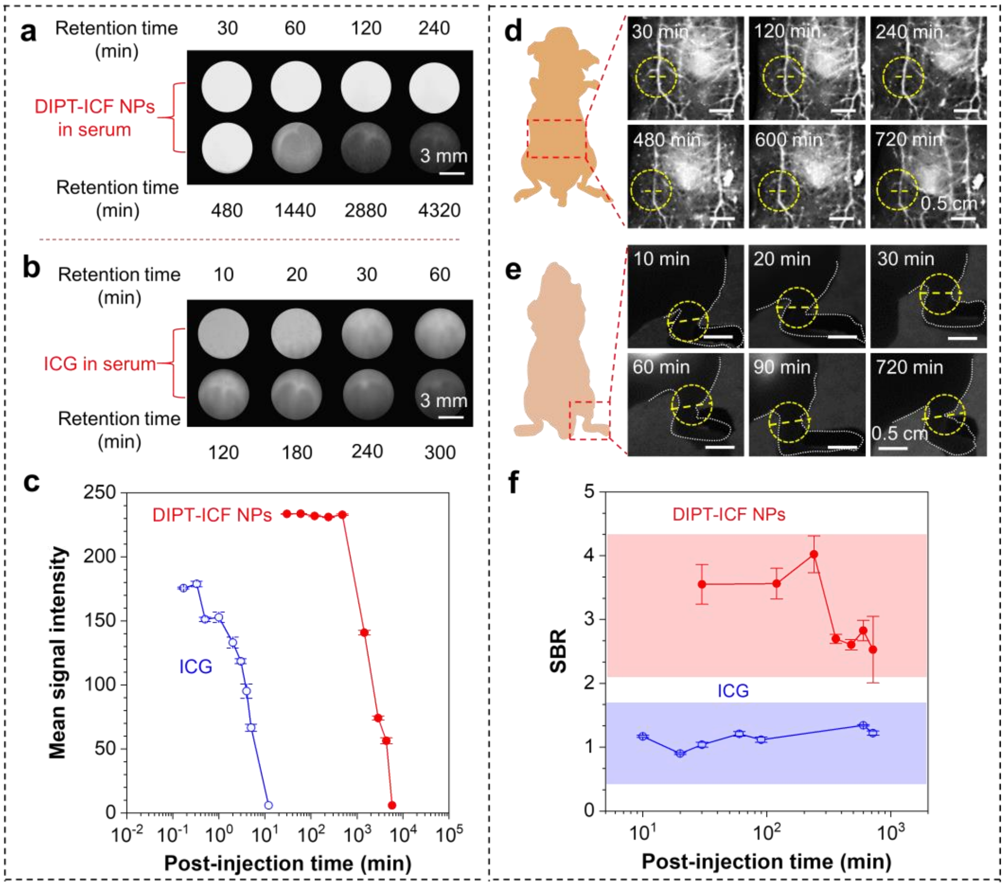

Figure S33. Retention time of DIPT-ICF NPs and ICG in the blood circulation of mice. (a) NIR-II images of serum collected from mice's blood were captured at different time points (30, 60, 120, 240, 480, 1440, 2880, and 4320 min) post-injection of DIPT-ICF NPs through tail veins (980 nm laser, 60 mW cm^{-2}, 1319 nm LP, and exposure time 200 ms). (b) NIR-I images of serum collected from mice at different time points (10, 20, 30, 60, 120, 180, 240, and 300 min) post-injection of ICG via tail veins were captured (808 nm laser, 900 nm LP, and exposure time 30 ms). (c) The quantitative analysis curves of mean signal intensity of DIPT-ICF NPs and ICG in panel (a) and (b), respectively. (d) The NIR-II angiography was performed on abdominal vessels of mice at different time points post-injection of DIPT-ICF NPs (980 nm laser, power density 60 mW cm^{-2}, 1319 nm LP, and exposure time 200 ms). (e) The NIR-I images were captured on mice at different time points after ICG injection (808 nm laser, 900 nm LP, and exposure time 35 ms). (f) The quantitative analysis of SBR in ROI regions marked with the yellow dashed lines and circles in (d) and (e).

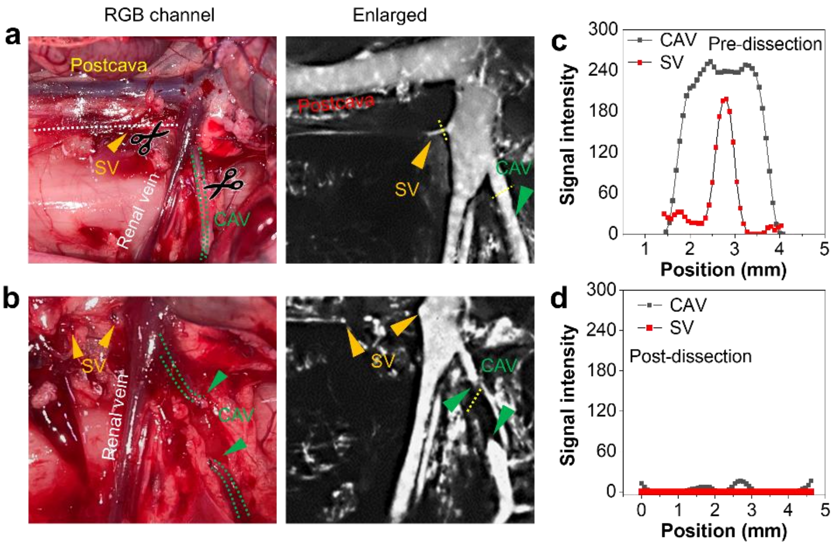

Figure S34. The renal vein branches were ligated and severed in image-guided donor nephrectomy. The DIPT-ICF NPs was used as the NIR-II contrast agent. NIR-II images were captured before (a) and after (b) renal vein branches ligating and severing. The white and green dashed lines represent the small vein (SV) and cranial abdominal vein (CAV) buried in the tissue, respectively. The orange arrows represent the ligation site of SV in panel (a) and two severed ends of SV in panel (b); The green arrows represent the ligation site of CAV in panel (a) and two severed ends of CAV in panel (b). (c, d) The cross-sectional fluorescence intensity profiles of SV and CAV along with the yellow dashed lines in panel (a) and (b), respectively.

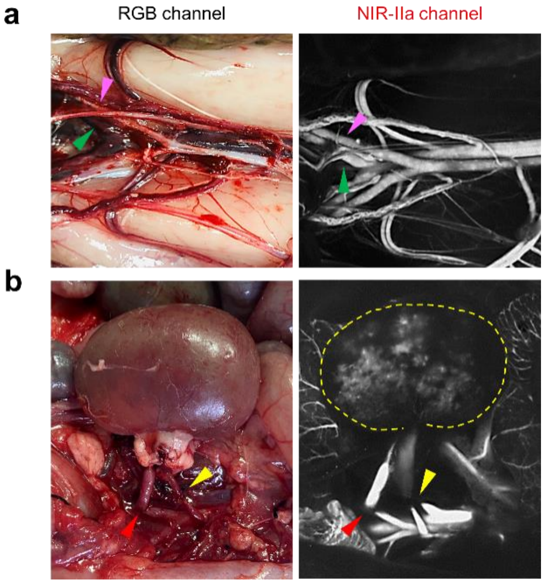

Figure S35. NIR-IIa fluorescence angiography of iliac vessels before and after heterotopic kidney transplantation. (a) Angiography before kidney transplantation. (b) Angiography after kidney transplantation. The white arrow represents the right common iliac artery, and the green arrow represents the right common iliac vein. The red arrow indicates the end-to-side anastomosis between the right renal artery and the

right common iliac artery, and the yellow arrow indicates the end-to-end anastomosis between the right renal artery and the right common iliac vein.

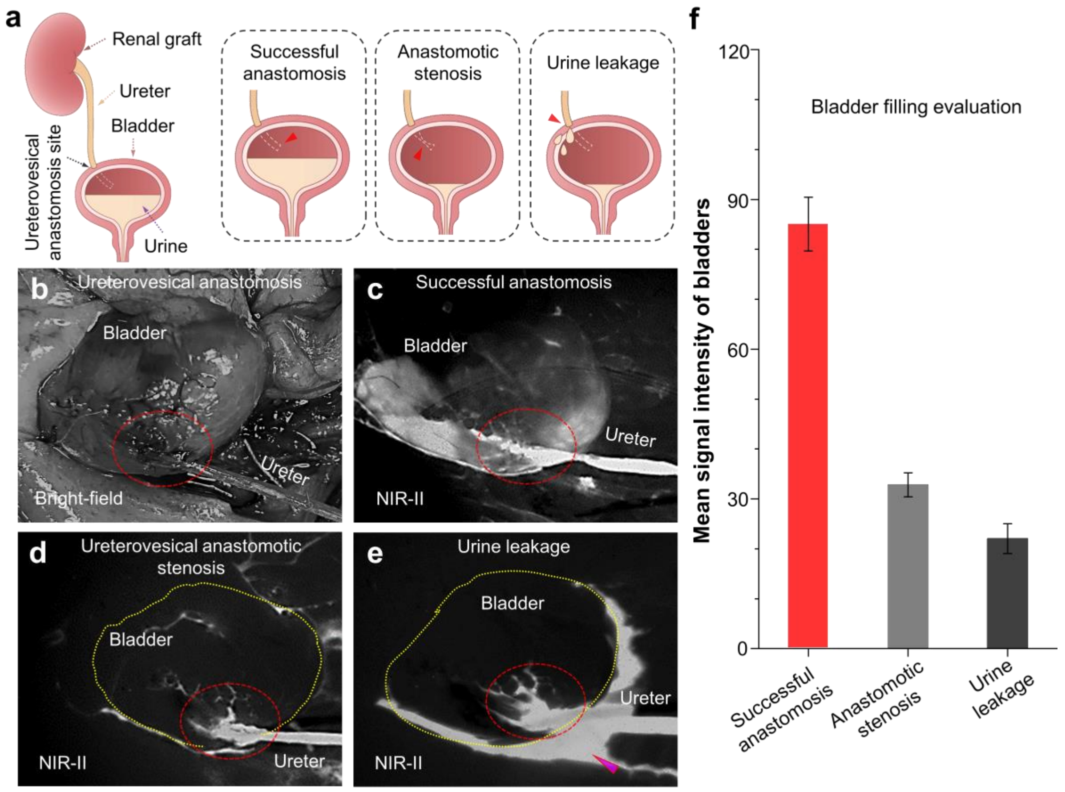

Figure S36. NIR-II anterograde ureterography after the upper urinary tract reconstruction during renal transplantation based on the nano-contrast agent of DIPT-ICF NPs. (a) A schematic of anterograde NIR-II ureterography for the diagnosis of ureterovesical anastomotic complications in kidney transplantation. The red arrows indicate ureterovesical anastomotic sites. The bright-field (b) and NIR-II fluorescence images (c) of successful ureterovesical anastomosis. NIR-II images of ureterovesical anastomotic abnormalities, such as ureterovesical anastomosis stricture (d) and urine leakage (e) were captured after ureterovesical anastomoses with the DIPT-ICF nano contrast agent. (f) Evaluation of urinary filling in bladders was carried out by the NIR-II imaging approach for panel (c), (d), and (e), respectively. The red dashed circles indicate ureterovesical anastomosis sites. The purple arrow in (e) represents the failing ureterovesical anastomosis, resulting in urine leakage. All NIR-II images were captured with a 980 nm laser at a power density of 60 mW cm^{-2}, a 1319 nm LP filter, and an exposure time of 200 ms.

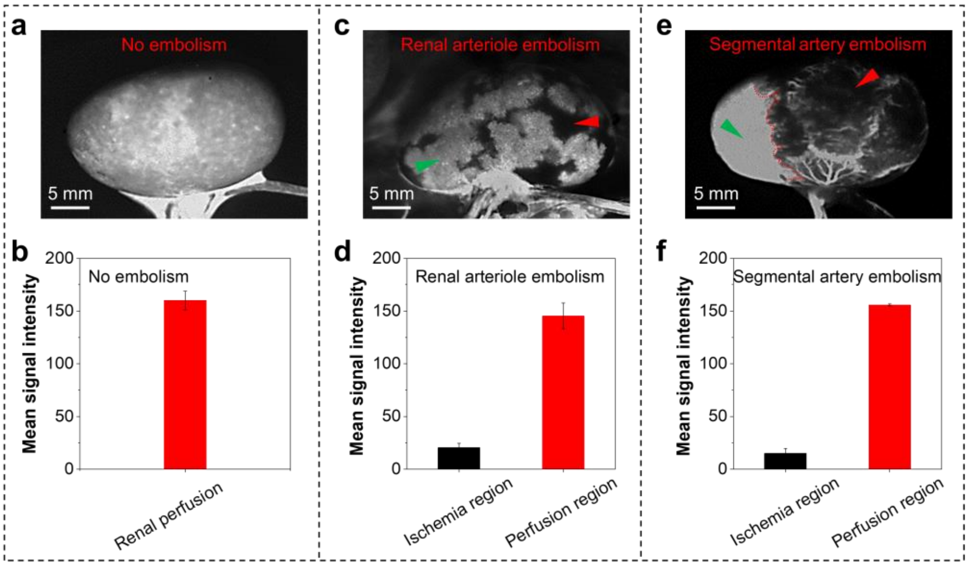

Figure S37. As a NIR-IIa fluorescence contrast agent, DIPT-ICF NPs were used to diagnose intrarenal vascular embolism. The NIR-II images of rabbit kidneys without thrombosis (a), with arteriole embolisms (c), and larger branches thrombosis (e). (b, d, f) Quantitative analysis of the mean NIR-II signal intensity on renal perfusion regions and ischemia regions in panel (a), (c), and (e), respectively. The green and red arrows represent perfusion regions and ischemia regions of model kidneys, respectively.

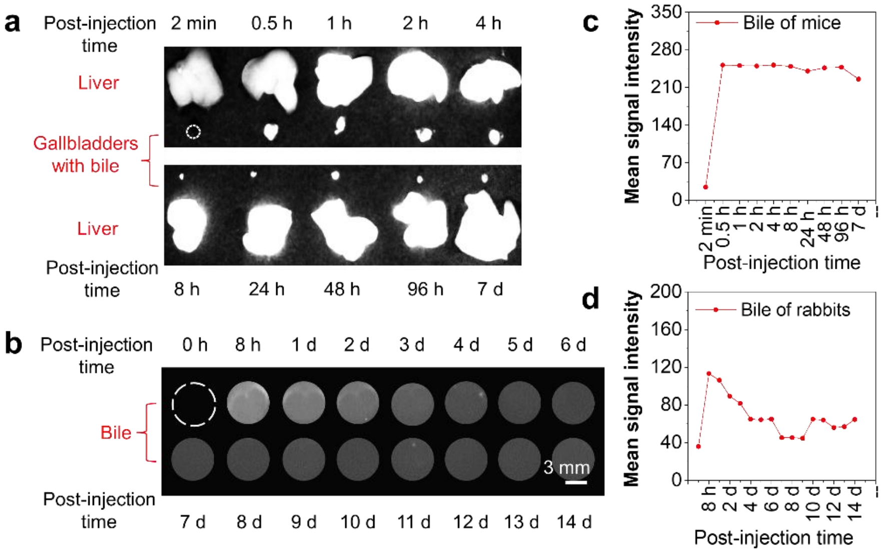

Figure S38. (a) NIR-II images of livers and gallbladders retrieved from the mice at different time points post DIPT-ICF NPs injection. (b) NIR-II images of the bile obtained from the rabbits at different time post DIPT-ICF NPs injection through ear margin vein. (c, d) Quantitative analysis of NIR-II signal intensity of bile in panel (a) and (b), respectively.

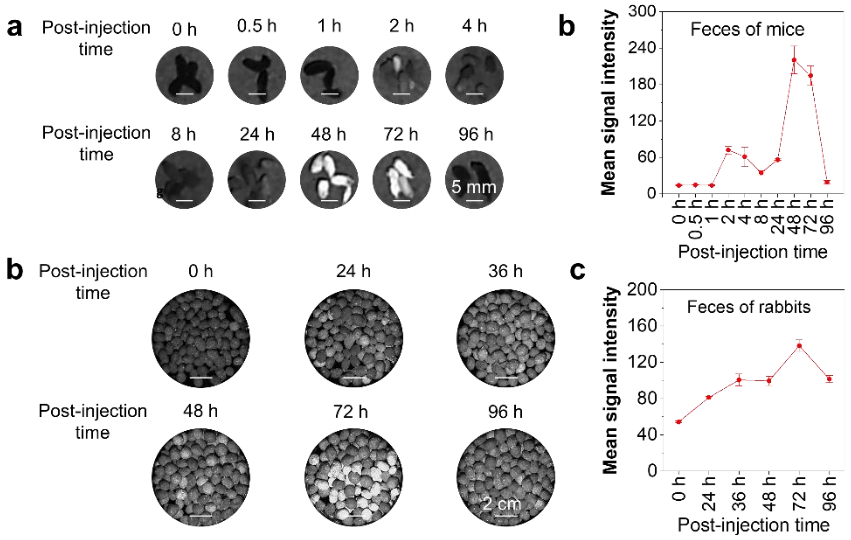

Figure S39. NIR-II images of feces collected from the mice (a) and rabbits (b) at different time points post DIPT-ICF NPs injection. (c, d) Quantitative analysis of NIR-II signal intensity of feces in panel (a) and (b), respectively.

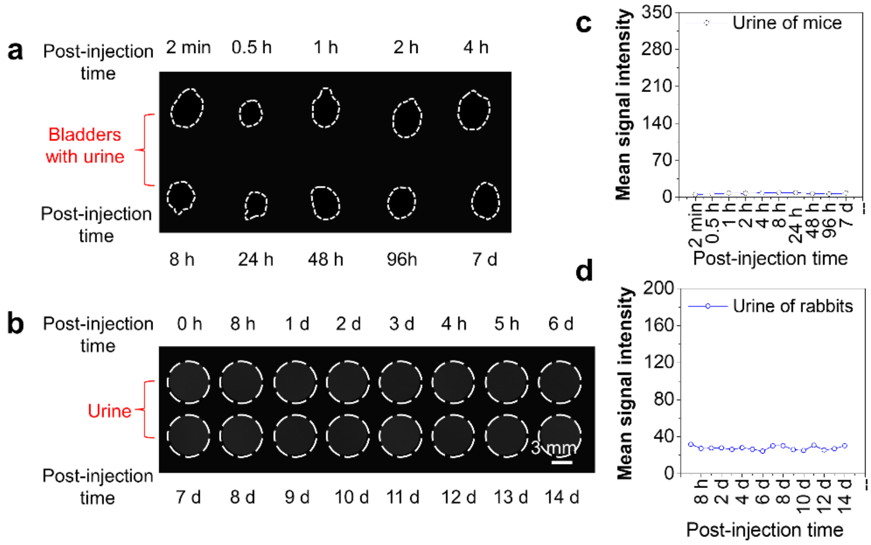

Figure S40. NIR-II images of the urine collected from the mice (a) and rabbits (b), at different times post DIPT-ICF NPs injection. (c, d) Quantitative analysis of NIR-II signal intensity of urine in panel (a) and (b), respectively.

Supplementary tables

Table S1. Optical properties of DIPT-IC, DIPT-ICF, IR-26 and IR-1061.

Dye	λ_{abs} (nm)	ε ($M^{-1}cm^{-1}$)	Absolute Φ (%)	Brightness $\varepsilon\Phi$ ($M^{-1}cm^{-1}$)
DIPT-IC	748	5.77×10^4	0.06	34.62
DIPT-ICF	818	1.79×10^5	0.09	161.1
IR-26	1080	1.49×10^5	0.05	74.5
IR-1061	1060	2.51×10^5	0.08	200.8
IR-26 NPs	1080	1.32×10^3	0	0
IR-1061 NPs	1061	1.03×10^4	0	0

Note: The absorbance is measured by a UV-Vis-NIR Spectrophotometer (Shimadzu, Japan). The absolute PLQYs of DIPT-IC, DIPT-ICF, and IR-1061 in the table measured by a Quantaurus-QY Plus UV-NIR (Hamamatsu, Japan). The absolute PLQY of IR-26 sources from the literature [6]. IR-26 and IR-1061 were coated by F127.

Table S2. Blood samples of mice were collected 43 days later after injection of DIPT-ICF NPs (1 mg mL^{-1}, 200 μL, experiment group) or 1 × PBS (200 μL, control group).

Hepatotoxicity	DIPT-ICF NPs			PBS		
	mean	SD[a]	mice	mean	SD[a]	mice
Alanine aminotransferase (U/L)	41.13	8.10	3	40.73	2.10	3
Aspartate Transaminase (U/L)	103.67	12.55	3	109.33	20.59	3
Total protein (g/L)	41.53	5	3	37.73	6.53	3
Albumin (g/L)	29.13	0.64	3	23.13	4.24	3
Albumin/globulin	2.6	1.07	3	2.12	1.47	3
Total bilirubin (umol/L)	3.46	0.86	3	1.18	0.19	3
Indirect bilirubin (umol/L)	2.15	0.64	3	0.83	1.03	3
**Nephrotoxicity**						
Blood urea nitrogen (mmol/L)	14.61	2.13	3	13.23	0.17	3
Serum creatinine (μmol/L)	3.2	2.2	3	3.0	0.53	3
Calcium (mmol/L)	2.06	0.21	3	1.68	0.06	3
Phosphorus (mmol/L)	2.15	1.99	3	3.24	1.74	3
**Haematological toxicity**						
White blood cells (10^9/L)	3.59	0.96	3	3.77	0.54	3
Red blood cells (10^{12}/L)	5.62	0.23	3	5.88	0.19	3
Hemoglobin (g/L)	137	1.35	3	140.33	4.04	3
Mean Corpuscular Volume (f L)	49.83	1.5	3	50.5	0.46	3
Platelets (10^9/L)	455.33	77.05	3	346	113.62	3

[a] Standard deviation

Table S3. Blood samples of rabbits were collected 43 days later after injection of DIPT-ICF NPs (1.5 mg kg^{-1}, experiment group) or 1 × PBS (200 μL, control group).

**Hepatotoxicity**	DIPT-ICF NPs mean	DIPT-ICF NPs SD[a]	DIPT-ICF NPs rabbits	PBS mean	PBS SD[a]	PBS rabbits
Alanine aminotransferase (U/L)	45.80	1.73	3	47.27	2.32	3
Aspartate Transaminase (U/L)	57.87	10.10	3	54.13	7.51	3
Total protein (g/L)	44.2	5.14	3	47.1	4.37	3
Albumin (g/L)	35.2	2.39	3	37.42	3.69	3
Albumin/globulin	4.76	1.57	3	5.18	0.83	3
Total bilirubin (umol/L)	0.66	0.29	3	0.57	0.19	3
Indirect bilirubin (umol/L)	0.22	0.08	3	0.17	0.07	3
**Nephrotoxicity**						
Blood urea nitrogen (mmol/L)	7.97	0.12	3	8.2	0.11	3
Serum creatinine (μmol/L)	90.97	1.16	3	108.1	2.05	3
Calcium (mmol/L)	3.37	0.2	3	3.54	0.45	3
Phosphorus (mmol/L)	1.12	0.01	3	2.76	0.1	3
**Haematological toxicity**						
White blood cells (10^9/L)	6.55	0.27	3	6.48	0.47	3
Red blood cells (10^{12}/L)	3.87	0.35	3	3.72	0.09	3
Hemoglobin (g/L)	120.67	9.81	3	116	2	3
Mean Corpuscular Volume (f L)	57.43	1.47	3	55.92	4.29	3
Platelets (10^9/L)	115.33	47.38	3	137	17.58	3

[a] Standard deviation

Reference

1. Williams ATR, Winfield SA, and Miller JN. Relative fluorescence quantum yields using a computer-controlled luminescence spectrometer. *Analyst* 1983; **108**: 1067-71.
2. Hong G, Zou Y, and Antaris AL et al. Ultrafast fluorescence imaging in vivo with conjugated polymer fluorophores in the second near-infrared window. *Nat Commun* 2014; **5**: 1-9.
3. Jacobsen IA. Renal transplantation in the rabbit: a model for preservation studies. *Lab Anim* 1978; **12**: 63-70.
4. Watson CJE, Friend PJ, and Marson LP. 11 - Surgical Techniques of Kidney Transplantation. In: Knechtle SJ, Marson LP, Morris PJ (eds.). Kidney Transplantation - Principles and Practice (Eighth Edition). Philadelphia: Elsevier; 2019. 157-72.
5. Deng G, Yin P, and Zeng X et al. Effects of the length and steric hindrance of π-bridge on molecular configuration and optoelectronic properties of di-indole [3,2-b:4,5-b'] pyrrole-based small molecules. *Dyes Pigments* 2019; **171**: 107687.
6. Semonin OE, Johnson JC, Luther JM et al. Absolute Photoluminescence Quantum Yields of IR-26 Dye, PbS, and PbSe Quantum Dots. *J. Phys. Chem. Lett.* 2010; **1**: 2445-50.
